# Computational investigation of sphingosine kinase 1 (SphK1) and calcium dependent ERK1/2 activation downstream of VEGFR2 in endothelial cells

**DOI:** 10.1371/journal.pcbi.1005332

**Published:** 2017-02-08

**Authors:** Hojjat Bazzazi, Aleksander S. Popel

**Affiliations:** Department of Biomedical Engineering, School of Medicine, Johns Hopkins University, Baltimore, Maryland, United States of America; University of Pennsylvania, UNITED STATES

## Abstract

Vascular endothelial growth factor (VEGF) is a powerful regulator of neovascularization. VEGF binding to its cognate receptor, VEGFR2, activates a number of signaling pathways including ERK1/2. Activation of ERK1/2 is experimentally shown to involve sphingosine kinase 1 (SphK1) activation and its calcium-dependent translocation downstream of ERK1/2. Here we construct a rule-based computational model of signaling downstream of VEGFR2, by including SphK1 and calcium positive feedback mechanisms, and investigate their consequences on ERK1/2 activation. The model predicts the existence of VEGF threshold in ERK1/2 activation that can be continuously tuned by cellular concentrations of SphK1 and sphingosine 1 phosphate (S1P). The computer model also predicts powerful effects of perturbations in plasma and ER calcium pump rates and the current through the CRAC channels on ERK1/2 activation dynamics, highlighting the critical role of intracellular calcium in shaping the pERK1/2 signal. The model is then utilized to simulate anti-angiogenic therapeutic interventions targeting VEGFR2-ERK1/2 axis. Simulations indicate that monotherapies that exclusively target VEGFR2 phosphorylation, VEGF, or VEGFR2 are ineffective in shutting down signaling to ERK1/2. By simulating therapeutic strategies that target multiple nodes of the pathway such as Raf and SphK1, we conclude that combination therapy should be much more effective in blocking VEGF signaling to EKR1/2. The model has important implications for interventions that target signaling pathways in angiogenesis relevant to cancer, vascular diseases, and wound healing.

## Author summary

Vascular endothelial growth factor (VEGF) signaling is a potent regulator of angiogenesis, the growth and development of new vessels out of a preexisting vascular network. Angiogenesis requires enhanced survival, proliferation, and motility of the vascular endothelial cells. Crucial signaling endpoints in VEGF-mediated angiogenic response include elevation in intracellular calcium and the activation of the proteins ERK1 and 2 (ERK1/2). In this study, we have developed a novel computer model for the activation of ERK1/2 and calcium downstream of VEGF receptor type 2 (VEGFR2). Our model is the first of its kind to incorporate and investigate the consequences of calcium elevation and the role of a cellular lipid modifier known as sphingosine kinase 1 (SphK1). We also utilize the model to simulate therapeutic strategies targeting VEGF signaling to ERK1/2 indicating inefficiency of single therapies known as tyrosine kinase inhibitors (TKI) that target receptor phosphorylation. Computer simulations indicate that combination therapy is essential for effective blockade of this important pathway. Our results have important implications for human diseases such as cancer where plethora of anti-VEGF therapies are currently employed. Overall, our computer model sheds new light on a complex feedback involving SphK1 and calcium that radically alters the response of cells to VEGF.

## Introduction

Angiogenesis is the growth of new capillaries from the pre-existing vasculature. The process of angiogenesis involves increased proliferation, survival, and migration of the endothelial cells that form the foundation of a developing vascular bed [1]. This process is critically involved in both health and disease [2]. Physiologically, it is involved in placental vascularization during pregnancy and the growth of normal blood vessels during development. Pathological angiogenesis is crucial in vascularizing tumors, a critical step in transition to neoplasm and cancer [3]. Newly formed tumor vasculature also contributes to the process of metastasis by shedding tumor cells into the bloodstream that then travel throughout the body and provide seeds for new tumors in more distant tissues [4,5]. In diseases such as age-related macular degeneration and diabetic macular edema, angiogenesis contributes to the neovascularization of the retina and the leakiness of the ocular blood vessels that may eventually lead to blindness [6]. In other diseases such as peripheral arterial disease, the opposite occurs where the blood capillaries and vessels regress leading to the reduction and, in some cases, total cessation of the blood flow to lower extremities [7]. Left untreated, this condition may require amputation of the regions affected by the lack of blood flow.

Considering the crucial role of angiogenesis in human health and disease it is no wonder that there is deep interest in understanding the mechanisms responsible for regulation and modulation of this phenomenon. Several endothelial cell growth factors have been identified as being critical for priming the endothelial cells to undergo the processes that would eventually lead to the generation of new blood vessels. One critical factor is the vascular endothelial growth factor A (VEGF-A, hereby referred to as VEGF) identified as a potent inducer and regulator of angiogenesis [8]. There are six different human isoforms of VEGF, with VEGF_165_ being by far the most intensely studied member of the group. VEGF_165_ sits at the helm of signaling pathways that prominently include VEGF receptor 2 (VEGFR2), VEGF receptor 1 (VEGFR1), and neuropilin-1 and 2 (NRP1 and NRP2) co-receptors. VEGF signaling is initiated by the binding of VEGF to VEGFR2 with subsequent VEGFR2 auto-phosphorylation on several tyrosine residues, leading to pro-angiogenic phenotypes such as increased cell proliferation and motility. Binding of VEGF to VEGFR2 and subsequent dimerization and auto-phosphorylation of at least six tyrosine residues (with Y1175 being the most widely studied) on the receptor leading to the recruitment of various adaptor proteins that transduce the phospho-tyrosine signal to downstream pathways including PI3K/AKT, Nitric Oxide (NO), and ERK1/2 that play crucial roles in determining and regulating vascular function [9]. Of different pathways, ERK1/2 activation has been shown to play a major role in VEGF-induced angiogenesis by inducing endothelial cell proliferation and motility [10–13]. The canonical MAPK pathway that leads to ERK1/2 phosphorylation, involves the recruitment and binding of the adaptor proteins Grb2 and SOS to the phospho-tyrosine sites on the receptors that activate small G protein Ras. Activated Ras then binds and activates Raf with subsequent activation of MEK1/2 and ERK1/2, with the eventual nuclear translocation of activated ERK1/2 [14,15]. While this canonical pathway operates in the activation of ERK1/2 in response to other growth factors such as the fibroblast growth factor (FGF) and epidermal growth factor (EGF), VEGF activation of this cascade seems to be fundamentally different involving the complex feedback mechanism initiated by calcium and ERK1/2-dependent activation of sphingosine kinase 1 (SphK1) [9,11,16,17]. Given the enormous complexity of these signaling pathways, computational models have been developed to aid in the elucidation of basic mechanisms of signal transduction and identify nodes of the pathway that might act as hubs in modulating the strength and duration of the signal. There is a large literature of mathematical models focusing on the quantitative understanding of the canonical MAPK pathway initiated by the activation of ErbB [18,19], EGFR [20,21], and VEGFR2 [22,23]. While ErbB and EGFR do signal via the canonical MAPK pathway to ERK, current experimental data on the VEGFR2 signaling suggest that the signaling is through a mechanism involving SphK1 and calcium [11,24]. Shu *et al*. were the first to show that the inhibition of SphK1 and PKC completely abolished the pERK1/2 signal [11]. Subsequent evidence suggested that the activation of SphK1 is through phosphorylation by activated ERK1/2 [16]. The mechanism becomes more complicated considering that activated SphK1 needs to be translocated from the cytoplasm to the plasma membrane, and that this is mediated by calcium binding to calcium- and integrin binding protein 1 (CIB1) [25].

Our intention here is to provide the first proof-of-principle simulations for the activation of ERK1/2 by SphK1 and calcium, and investigate the consequences of SphK1 positive feedback on VEGF signal transduction to ERK1/2. In so doing, we also develop a model that includes the major VEGF binding receptors on the cell surface: VEGFR1, VEGFR2, and NRP1. We also include the effect of internalization and degradation of the receptors by considering signaling from separate endocytic and membrane compartments. While internalization has been incorporated in recent computational models of VEGFR2 signaling [22,26], our model is the first to explicitly incorporate multi-complex internalization and signaling to downstream targets such as ERK1/2 and calcium.

Our basic assumption based on the available evidence is that SphK1 signaling is sufficient for activation and sustenance of ERK1/2 downstream of VEGFR2. Moreover, by explicitly incorporating a mechanistic model of cytoplasmic and ER calcium dynamics in the VEGF model here, we highlight the important connections between calcium dynamics and ERK1/2 activation.

Regarding the computational implementation of the model, a radical departure from the existing models of VEGF signaling, is the application of a rule-based modeling approach utilizing the programming language BioNetGen to accurately capture all the species and their interactions in the cell [27,28]. This method has been successfully applied to develop a detailed model of EGF/EGFR signaling taking into account the combinatorial complexity generated by multi-domain protein interactions [29]. BioNetGen automatically generates the biochemical network given the input rules operating on the seed species for domain interactions and phosphorylation reactions. This methodology has the added advantage of including receptor complexes and single or doubly phosphorylated species. While this can be done with conventional modeling where the reaction list is written down manually *a priori*, rule-based approach generates all the relevant species and bypasses the potential for errors inherent in manual construction of the pathway.

## Results

### Rule-based model construction for SphK1 dependent ERK1/2 activation downstream of VEGF

**Fig 1:**
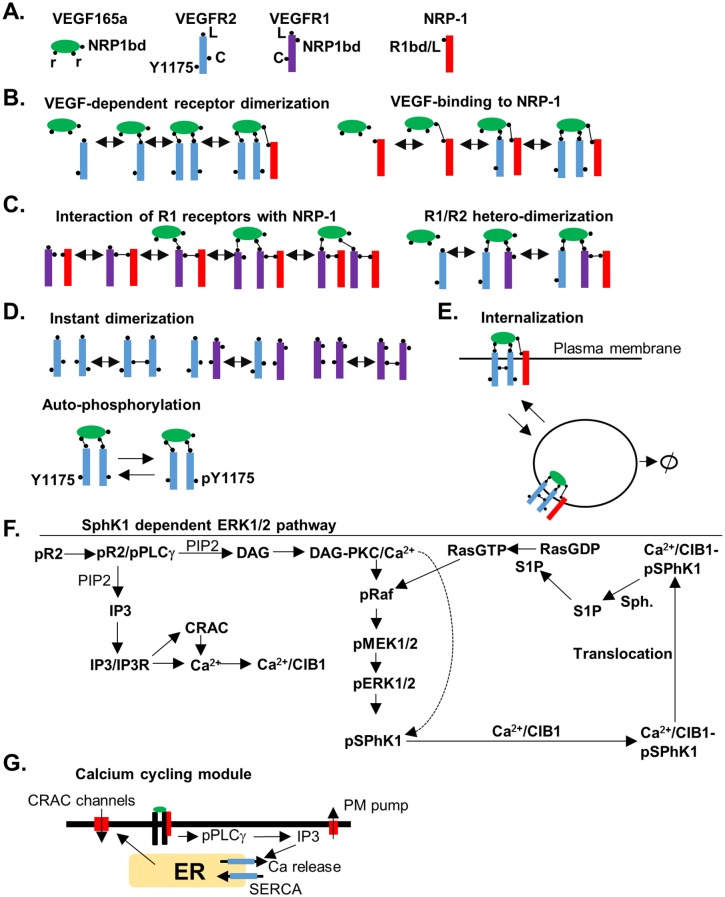
Rules for constructing the SphK-1 dependent ERK1/2 pathway. **A**. Species with domain structures labeled. VEGF with three binding sites, two for binding to the receptors and a C-terminal binding domain for binding to NRP1. VEGFR2, with ligand binding site (L), receptor coupling site (C), and the tyrosine in the position 1175 (Y1175), VEGFR1 with ligand binding domain (L), receptor coupling site (C), and the NRP1 binding site (NRP1bd). NRP1 with the ligand binding site and the VEGFR2 binding site labeled (R1bd/L), **B**. The rule for the interaction of VEGF and VEGFR and subsequent dimerization included, **C**. The rule for ligand-independent interaction of VEGFR1 and NRP1, **D**. Ligand-independent dimerization of the receptors through the coupling sites (top). Ligand-dependent phosphorylation of the Y1175 on VEGFR2 (bottom), **E**. Receptor-complexes undergo internalization and degradation from the plasma membrane, **F**. Signaling pathway for the activation of ERK1/2 downstream of phosphorylated Y1175 (pY1175), **G**. Elements of the calcium-cycling module of the model. Activated PLCγ results in the generation of IP3 that then diffuses and binds to and activates IP3 sensitive calcium release channels on the ER membrane. The decline in ER calcium concentration results in the opening of the calcium release activated calcium (CRAC) channels.

In the rule-based modeling approach, the protein domains and modification sites are explicitly included in the model design process and the rules for the modification and binding are implemented using a programming environment such as BioNetGen (see the supplementary material for the BioNetGen file, the corresponding SBML file, and the list of parameters with their descriptions). The general framework for our rule-based model and the constructed signaling pathway are summarized in Fig 1.

Fig 1A shows different binding sites for the initial receptor species in the model. VEGF contains three binding sites: two that are capable of binding to single binding sites on VEGFR1 and VEGFR2, and the third binding site is located on C-terminal domain that binds NRP1 [30]. VEGFR2 has two binding sites, one for VEGF and the other for ligand-independent coupling with another VEGFR2 or VEGFR1 molecule [31]. Also included, is the essential phosphotyrosine site that can be modified by phosphorylation and de-phosphorylation. VEGFR1 has a binding site for the ligand and a ligand-independent coupling site with VEGFR2 or VEGFR1 as shown. Ligand-independent receptor dimerization of VEGFR2 and VEGFR1 has been observed experimentally and have been incorporated in a recent computational model [32]. VEGFR1 also includes a ligand-independent binding site to NRP1. NRP1 has a single binding site that can competitively bind either VEGF or VEGFR1 [33]. In the absence of the ligand and VEGFR1, NRP1 is assumed to be in monomeric form. VEGF-dependent dimerization of VEGFR2 proceeds by the rule shown in Fig 1B. This rule is capable of generating multi-receptor complexes by including VEGFR2 and NRP1 in the complex. VEGF can also bind to NRP1 binding site directly from the solution. The interaction rule for the binding of VEGFR1 to NRP1 is illustrated by Fig 1C. VEGFR1 and VEGFR2 can also heterodimerize by VEGF according to the scheme shown. Ligand-independent dimerization of the receptors can also occur (Fig 1D). This can generate not only homodimers of VEGFR2/VEGFR2 and VEGFR1/VEGFR1, but also VEGFR1/VEGFR2 heterodimers. Note that all the species interact simultaneously according to the rules that take into account the combinatorial complexity inherent in the interacting multi-domain proteins. VEGFR2 receptors can undergo auto-phosphorylation if they are part of a homodimer complex with the ligand (Fig 1D). We also include internalization of the receptor complexes as shown in Fig 1E. In the model, we also include internalization and degradation of the receptors in the absence of ligand, constrained by the condition that the number of receptors in the absence of ligand remain steady during simulations.

Based on current evidence from the literature combined with the VEGF pathway information from the Reactome database [34,35], a pathway from activated VEGFR2 receptors to ERK1/2 activation is constructed as shown in Fig 1F. According to the experimental evidence, SphK1 is phosphorylated and activated mainly by pERK2 [16]. Direct phosphorylation of SphK1 by active PKC is included in the diagram for completeness and can augment the effects through ERK1/2, but the experimental evidence for direct activation of SphK1 by PKC within the context of VEGF signaling to ERK1/2 is not adequate and thus is not explicitly incorporated in the model [16]. Once activated, SphK1 is translocated to the plasma membrane by the calcium and integrin binding protein 1 (CIB1). CIB1 has a myristoyl switch that is activated upon calcium binding [25]. Calcium/CIB1/phospho-SphK1 complex is translocated to the plasma membrane employing the myristoylated CIB1. SphK1 then phosphorylates its substrate, sphingosine (Sph), generating the diffusible sphingosine 1 phosphate (S1P). S1P then activates Ras in a process conjectured to involve the inhibition of a Ras GTPase activating protein (RasGAP).

A significant addition to the model is the inclusion of a detailed calcium cycling module illustrated in Fig 1G. This module includes the calcium release activated calcium channels (CRAC channels) that are crucial for VEGF-dependent rise in calcium [36,37]. Further details of the model are included in the supplementary section of this paper.

### Model parameterization

To estimate the parameters for receptor dynamics, the model is fitted to the total VEGFR2 levels using the data from [38,39]. All the relevant parameters are simultaneously fitted to a consistent set of experimental data. The variables computed by the model and the corresponding data are normalized to the maximum values.

**Fig 2:**
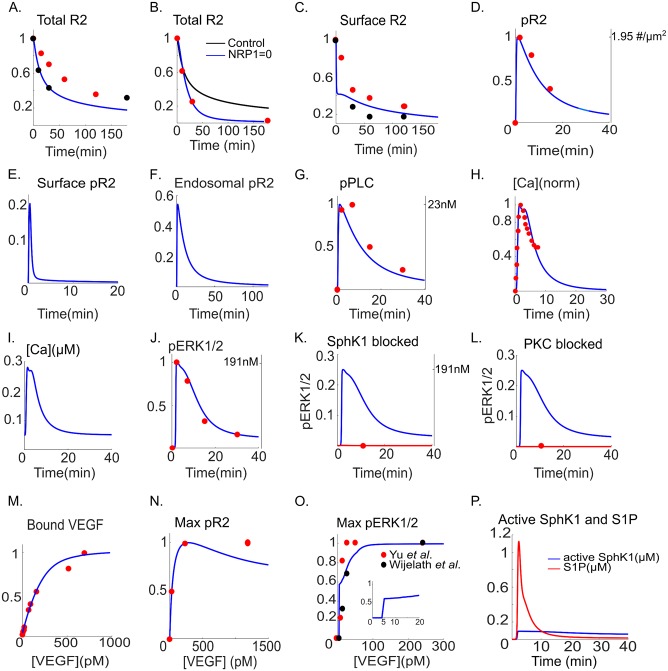
Model fit to the experimental data. **A**. Total VEGFR2 fraction from the model (blue, solid) fitted to the experimental data in [38] (red circles) and [39] (black circles), **B**. Total VEGFR2 level in the absence of NRP1 from the model (blue) is fitted to the experimental data in [39] (black circles). The control curve from the model (in the presence of NRP1) is shown (black), **C**. Surface VEGFR2 fraction from the model (blue) is fitted to the experimental data from [40] (red circles) and [38] (black circles), **D**. Normalized total phosphorylated VEGFR2 from the model is fitted to the data of Chabot *et al*. [41], **E**. Predicted fractional surface level of pVEGFR2 from the model, **F**. Predicted endosomal pVEGFR2, **G**. pPLCγ from the model is fitted to the normalized experimental data from [41] (red circles), **H**. Normalized calcium transient from the model (blue) fitted to the data in [36] (red circles), **I**. Raw calcium transient from the model, **J**. Phosphorylated pERK1/2 from the model is fitted to the experimental data from [41] (red circles), **K**. Inhibiting SphK1 in the model (red line) blocks ERK1/2 activation. Experimental data point from endothelial cells at t = 10 min is also shown [11] (red circle). Control pERK1/2 curve is also shown (blue), **L**. Inhibiting PKC blocks ERK1/2 activation in the model (red line) with the experimental data point at t = 10 min shown [11] (red circle). Control pERK12 is shown (blue), **M**. Bound VEGF from the model (blue) is fitted to the experimental data from [42] (red circles), **N**. Dose response curve for pVEGFR2 from the model (blue) fitted to the data in [43] (red circle), **O**. Maximum value of pERK1/2 from the model (blue) versus VEGF concentrations fitted to the experimental data in [44] (black circles) and [45] (red circles), **P**. Activated SphK1(blue) and S1P (red).

After 180 min of VEGF application, 80% of the receptors in the cell are lost as shown in Fig 2A.

We assume that VEGFR1 remains on the cell membrane and does not internalize. This is in accordance with experimental evidence indicating that VEGFR1 internalization requires VEGFR1 phosphorylation [46], and given the weak VEGFR1 auto-phosphorylation under normal conditions, it is reasonable to assume a constant VEGFR1 surface level. To include the effects of NRP1 on VEGFR2 level, Fig 2B shows the result of fitting the total VEGFR2 in the absence of NRP1 (NRP1 = 0 in the model) to the experimental data [39] (red, solid circles) in endothelial cells. In the absence of NRP1, levels of VEGFR2 decline to zero, while the control cell still retains 20% of its VEGFR2 content after 180 min. Surface VEGFR2 levels from the model (Fig 2C, blue) are fitted to two sets of experimental data that have utilized either flow cytometry [40] (solid circles, black) or western blot [38] (solid circles, red). According to the model, the effect of NRP1 association with VEGFR2 is to substantially increase the internalization rate (0.404 s^-1^ vs. 6.1×10^−2^ s^-1^) and the recycling rate to the plasma membrane (0.756 s^-1^ vs. 1.24×10^-3^s^-1^). While in this model the internalized receptors with NRP1 have higher degradation rate than receptors without NRP1 (1.18×10^-2^s^-1^ vs. 1.41×10^−3^ s^-1^), the combined effect of receptor internalization, recycling, and degradation, is the rapid decline in receptor number in the absence of NRP1 compared to the control (Fig 2B), consistent with the literature. Further, the recycling of the phosphorylated receptors is negligible in the model consistent with data in [39]. To constrain the signaling parameters we use the western blot measurements for pY1175, pPLCγ, and pERK1/2 [41]. We also simultaneously apply the constraint that blocking SphK1 abolishes pERK1/2 consistent with the experimental data in endothelial cells demonstrating that ERK1/2 activation is blocked at t = 10 min following SphK1 inhibition [11]. The phosphorylated pY1175 (normalized by total VEGFR2) from the model is fitted to the experimental data (solid red circles) (Fig 2D). The predicted fractional phosphorylated receptor levels (fraction of total receptors) at the surface and endosomal compartment are shown in Fig 2E and 2F. The surface receptors show rapid transient activation that declines to zero in 10 min. The endosomal signaling is sustained for ~40 min demonstrating that the internalized receptors are the major contributors to the sustenance of the VEGFR2 signal consistent with the current experimental and computational evidence [26,47]. The predicted value for the dephosphorylation of the receptors at the surface is ~150 fold higher than for the internalized receptors. It is instructive to note that this differential signaling from internalized receptors is an emergent property of the model and is not assumed *a priori*. Phosphorylated PLCγ is fitted to the experimental data (Fig 2G) showing a transient activation and decline of the signal in ~40 min. To find the parameters for calcium cycling module in the model, Fig 2H shows the normalized calcium signal from the model fitted to the normalized experimental data in [36]. The amplitude of the calcium transient was constrained to be within 200–300 nM in accordance with the empirical measurements [48]. The raw calcium transient is shown in Fig 2I with maximum calcium concentration of ~280nM reached in ~1.5 min with duration of ~20 min. While there is variation in the amplitude and duration of the calcium transients in response to different VEGF dosing strategies [49], the simulated calcium output from the model is consistent with the experimental data.

There are several parameters (S1 Table) for the activation of ERK1/2 including the parameters that define the strength of the positive feedback loop from SphK1. The data used to estimate these parameters are the time-course of pERK1/2 [41], the experimental data in human umblical vein endothelial cells (HUVEC) indicating that SphK1 and PKC inhibition block ERK1/2 activation [11], and the VEGF dose-response for ERK1/2 activation in HUVEC [44] and porcine aortic endothelial cells (PAEC) [45]. The fit of pERK1/2 from the model to the data is shown in Fig 2J, along with the constrain that blocking SphK1 blocks pERK1/2 (Fig 2K). PKC inhibition in the model abolishes pERK1/2 consistent with data (Fig 2L). To constrain receptor binding and coupling parameters, the binding curve for VEGF is computed under the condition that there is no receptor internalization (solid blue curve, Fig 2M) and is fitted to the data (red circles, Fig 2M) [42]. The VEGF dose response curve for pVEGFR2 is also constrained (Fig 2N, solid red circles) demonstrating half-maximal activation (EC50) at 30 pM consistent with experimental observation [43]. We were able to constrain the dose response curve for pERK1/2 using the two sets of experimental data in PAEC [45] and HUVEC [44]. The data demonstrated *in vitro* ERK1/2 activation in response to soluble VEGF at concentrations as low as 0.25 ng/ml (6 pM) in PAEC with ERK1/2 activation saturated at 1 ng/ml (24 pM) (Fig 2O, solid red circles). In HUVEC, the experimental data (Fig 2O, solid black circles) indicated that soluble VEGF is capable of activating ERK1/2 at concentrations as low as 0.5 ng/ml (12 pM). The maximum fractional pERK1/2 from the model is fitted to the data as shown in Fig 2O (solid blue line). The model predicts a threshold behavior in ERK1/2 in response to VEGF (2O, inset). For concentrations of VEGF below 5 pM, the ERK1/2 is incapable of being activated, while for values above 5 pM, there is a gradual increase in maximum pERK1/2 versus VEGF. Two important observations regarding the pERK1/2 dose-response curve are worth noting. First, the EC50 for pERK1/2 is lower than pVEGFR2 (~5pM for pERK1/2 and ~30pM for pVEGFR2). Second, constraining the pERK1/2 VEGF dose response using the available data predicts the existence of a threshold behavior at VEGF concentration of 5 pM that will be explored in more detail later. The threshold behavior (or bi-stable behavior using the language of dynamic systems theory) is expected in systems containing positive-feedback loops [50].

ERK1/2 signals are expected to exhibit wide range of durations and amplitudes [22]. An important aspect of the current study is to identify what parameters or mechanisms determine and modulate the duration and amplitude of the pERK1/2 signal given the positive feedback generated by SphK1 and calcium.

The predicted S1P reaches 1 μM after t~2.3 min and declines to baseline in ~20 min. The activated SphK1 signal is more sustained even after 40 min retaining a ~92 nM concentration (Fig 2P). The predicted curves for active PKC, active Ras, active Raf, and the dose-response curve for active Ras are included in S1 Fig. Active Ras also shows threshold behavior in response to VEGF at 5 pM.

In the next section, we apply global sensitivity analysis to better understand the effect of Sphk1 in shaping the pERK1/2 signal. We will also investigate other parameters modulating the threshold behavior of ERK1/2 activation in response to VEGF.

### Global sensitivity analysis and threshold behavior in ERK1/2 activation by VEGF

**Fig 3:**
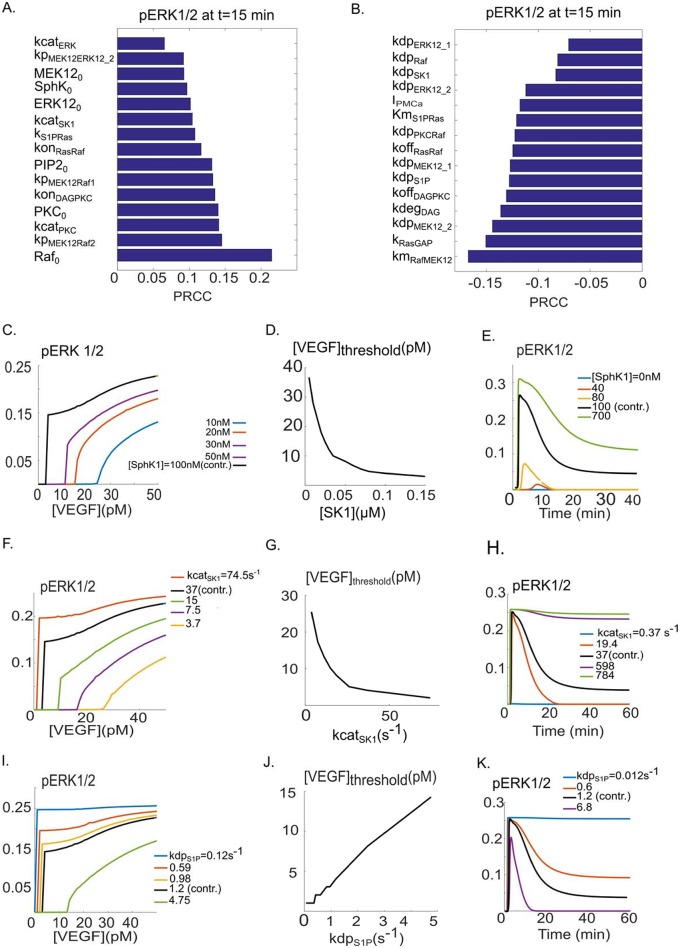
Global sensitivity analysis and VEGF threshold response of pERK1/2. **A**. Top 15 parameters with positive correlation according to the PRCC method. The top parameter is the total concentration of Raf. **B**. Top 15 parameters with negative correlation according to PRCC. The Michaelis-Menten-type parameter for the activation of MEK1/2 by Raf exhibits the highest negative correlation. **C**. Response of maximum fractional pERK1/2 relative to VEGF, with different SphK1 concentrations. The threshold value of VEGF is lowered in response to increasing SphK1 levels. **D**. VEGF threshold value is a decreasing function of the SphK1 concentrations, monotonically decreasing relative to SphK1. **E**. Sample curves for the time course of pERK1/2 for different values of SphK1 concentration. **F**. SphK1 catalytic rate modulates the VEGF threshold for ERK1/2 activation. **G**. There is a monotonically decreasing relation between the threshold value of VEGF and kcat_SK1_. **H**. Sample pERK1/2 versus time curves for different values of kcat_SK1_. **I**. The effect of the strength of S1P dephosphorylation on the threshold value of VEGF. **J**. VEGF threshold increases in response to increasing dephosphorylation rate of S1P, indicating an approximately linear response. **K**. Sample curves of pERK1/2 versus time in response to different values of kdpS1P.

We next carried out global sensitivity analysis to identify the most sensitive parameters influencing ERK1/2 activation. We utilized partial rank correlation coefficient (PRCC) [51] for this task which determines positive or negative monotonic relationships between the input parameters and the output observable (pERK1/2 in this case). Fig 3A shows the top 15 parameters with positive monotonic relationship with pERK1/2 at t = 15 minutes. PRCC coefficients were computed at 1, 2, 5 and 15 minutes.

We selected t = 15 minutes to identify parameters affecting the decaying and the plateau phase of pERK1/2. The top parameters include either the total protein levels or the parameters determining the kinetic rates. The partial rank correlation coefficient (PRCC) values for parameters with negative correlation to pERK1/2 are shown in Fig 3B. These parameters describe either the off kinetics of binding, the dephosphorylation rate, or the Michaelis-Menten-type constants for the phosphorylation reactions. We next applied the insights gained from sensitivity analysis to investigate the factors determining ERK1/2 response to VEGF ligand. Specifically, we investigated the possibility of modulating the threshold behavior observed by the model (previously illustrated in Fig 2O). Fig 3C shows the maximum value of pERK1/2 (normalized to the total ERK1/2 level) plotted against the concentration of VEGF for various values of total SphK1 concentrations. As illustrated by the figure, maximum pERK1/2 exhibits a threshold response for sufficiently high values of SphK1 concentrations. The threshold value for VEGF here is defined as the concentration of VEGF below which pERK1/2 is zero. The baseline model with 100 nM SphK1 results in a threshold value of 5 pM for VEGF. The threshold level increases gradually as the SphK1 concentration is lowered progressively to 10 nM. The increase in threshold value of VEGF is monotonic as shown in Fig 3D, from 36 pM ([SphK1_0] = 5 nM), to 3 pM ([SphK1_0] = 150 nM). The striking model prediction here is that a single parameter, namely the total SphK1 concentration, can have a significant effect on the sensitivity of the cell to VEGF by setting the threshold for ERK1/2 activation. Total SphK1 concentration is also a strong determinant of the amplitude and duration of pERK1/2 as illustrated by the pERK1/2 versus time curves for various values of the total concentration of SphK1 (Fig 3E). According to the model, SphK1 can modulate both the maximum and duration of the pERK1/2 signal. For sufficiently high SphK1, pERK1/2 signal reaches a plateau with very slow rate of decay (3E, green).

The strength of the SphK1 positive feedback is expected to be influenced by the catalytic rate of SphK1. This parameter was also a top hit in our global sensitivity analysis. The catalytic rate of SphK1 powerfully modulates the threshold for ERK1/2 activation as demonstrated by Fig 3F. The VEGF threshold value monotonically decreases as the catalytic rate increases as shown by Fig 3G. The threshold value of VEGF decreases from 25 pM to 2 pM, as the catalytic rate of SphK1 is increased from 3.7 s^-1^ to 74.5 s^-1^. The baseline fitted value of this parameter was kcat_SK1_ = 37.24 s^-1^ with the threshold value of VEGF = 5 pM. SphK1 catalytic rate also powerfully modulates the shape of the ERK1/2 activation signal as shown in Fig 3H. Once again, for sufficiently high catalytic rates, pERK1/2 signal reaches a plateau phase with no significant decay.

The responses to variations in total level of Raf are similar and are summarized in S2 Fig. To investigate the effect of a top negatively correlated parameter within the SphK1 feedback on pERK1/2 dynamics, Fig 3 (panels I and J) shows the effect of variations in the dephosphorylation rate of S1P (kdp_S1P_) on the threshold value of VEGF. Increasing kdp_S1P_, increases the VEGF threshold from 1 pM (kdp_S1P_ = 0.059 s^-1^) to 14 pM (kdp_S1P_ = 4.75 s^-1^). S1P dephosphorylation rate also crucially determines the maximum and duration of pERK1/2 signal (Fig 3K). Fig 3K shows the striking effect of decreasing kdp_S1P_ on pERK1/2 duration and plateau indicating that for sufficiently low values of this parameter, the pERK1/2 signal reaches a steady state with no decay (red and blue curves). Put together, these results demonstrate that ERK1/2 activation in response to VEGF is critically dependent on parameters affecting the SphK1 and S1P feedback downstream of phosphorylated receptors.

### Perturbations in calcium dynamics powerfully modulate ERK1/2 activation dynamics

**Fig 4:**
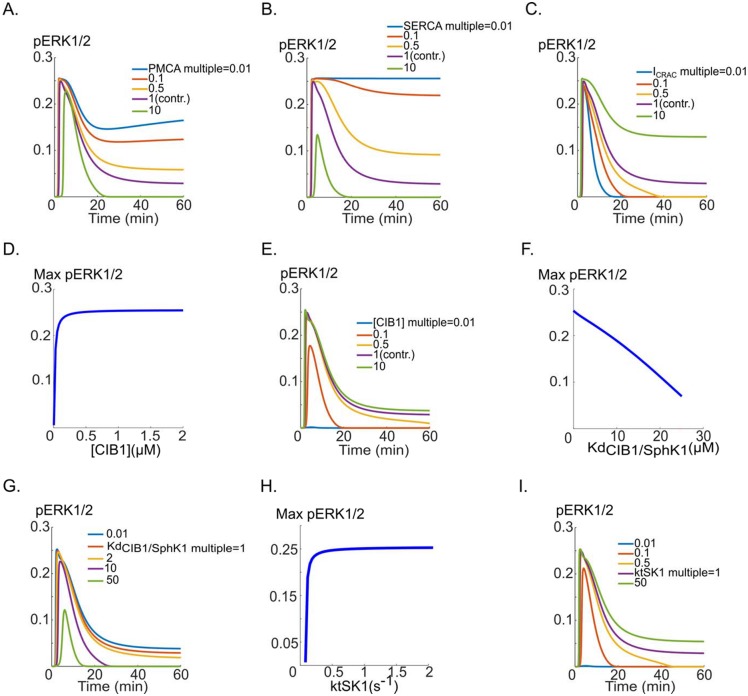
The effects of perturbations in calcium cycling on pERK1/2 dynamics. **A**. PMCA inhibition results in sustained pERK1/2 curve (blue), while the maximum value of pERK1/2 is unchanged. The plateau phase of pERK1/2 is progressively prolonged as the pump rate is reduced from the base value (pink, multiple of one), **B**. pERK1/2 versus time curves for five different values of the SERCA pump rate. **C**. The effect of varying CRAC channel current amplitude on the shape of the pERK1/2 versus time curve, illustrating the prolongation of the pERK1/2 signal, **D**. The changes in maximum pERK1/2 as a function of variations in total concentration of CIB1. There is rapid decline of pERK1/2 signal for CIB1 concentrations below ~80 nM. **E**. pERK1/2 versus time in response to CIB1 concentration, **F**. The dissociation constant for the binding of Ca^2+^/CIB1 to SphK1 starkly influences the maximum value of pERK1/2, **G**. pERK1/2 for different values of the dissociation constant, **H**. The maximum pERK1/2 as a function of the translocation rate of CIB1/SphK1 to the membrane (kt_SK1_), **I**. pERK1/2 versus time in response to changes in kt_SK1_.

Calcium signaling plays a crucial role in the activation of SphK1 by regulating CIB1-dependent SphK1 translocation to the membrane. Sensitivity analysis also identified the rate of membrane calcium pump (see Fig 2B) as being significant. Here we consider perturbations of calcium dynamics and present concrete and experimentally testable predictions of the model. The plasma membrane calcium pump (PMCA) rate significantly perturbs the duration of the ERK1/2 activation and is a sensitive determinant of the plateau phase of the signal as shown in Fig 4A.

**Fig 5:**
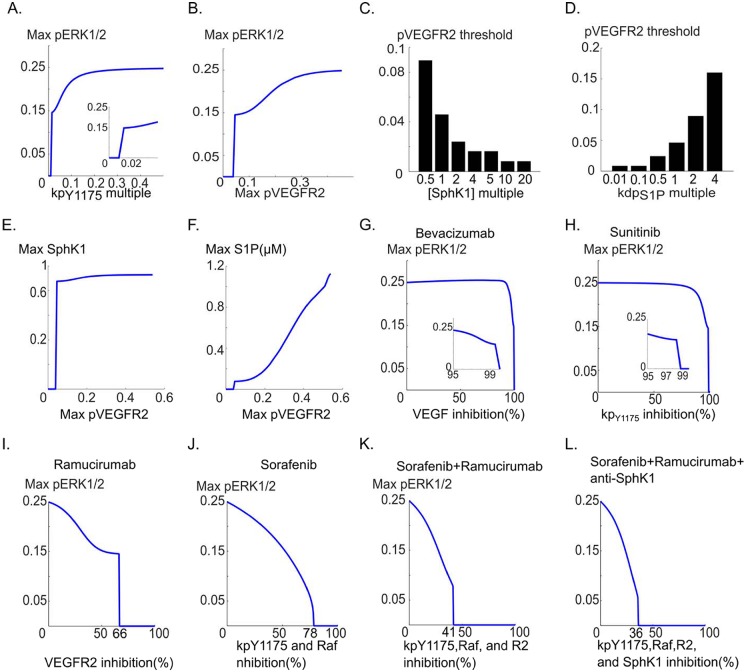
Relation between pERK1/2 and pVEGFR2 and its modulation by SphK1 pathway. **A**. pERK1/2 versus the phosphorylation rate of the receptor, **B**. Maximum pERK1/2 versus pVEGFR2 demonstrates the existence of a threshold value of pVEGFR2 below which there is no ERK1/2 activation. The critical value is 0.046, **C**. pVEGFR2 threshold is decreased in response to increases in SphK1 concentration. Increasing SphK1 to 1 μM from the baseline value of 0.1 μM reduces the threshold from 0.046 to 0.008, **D**. pVEGFR2 threshold value is sharply increased by increasing S1P dephosphorylation rate, from 0.008 to 0.16, **E**. Maximum active SphK1 versus max pVEGFR2 curve with similar threshold behavior, **F**. Maximum S1P shows monotonically increasing behavior relative to pVEGFR2 for pVEGFR2 values above the threshold value of 0.046, **G**. Maximum pERK1/2 versus percent inhibition of VEGF. The inset shows the threshold region: 99% sequestration of VEGF is necessary before ERK1/2 activation is blocked, **H**. Maximum pERK1/2 versus percent inhibition of the receptor phosphorylation. Inset shows the threshold region, **I**. Simulating the effect of VEGFR2 depletion with a mAb VEGFR2 inhibitor (e.g., ramucirumab) on pERK1/2, **J**. Simulating the effect of a generic multi-target tyrosine kinase inhibitor (e.g., sorafenib) on pERK1/2 simultaneously blocking VEGFR2 phosphorylation and Raf activation, **K**. Combined effect of sorafenib and ramucirumab demonstrating enhanced ERK1/2 inhibition, **L**. Combined effect of sorafenib, ramucirumab, and SphK1 inhibition on pERK1/2.

Blocking PMCA is predicted to convert a transient pERK1/2 signal into a plateau-phase response (Fig 4A, green to blue). Inhibition of ER calcium pump (SERCA) is considered next. Increasing the pump rate powerfully influences the shape of the pERK1/2 signal (Fig 4B). Similar to PMCA, sufficient inhibition of the SERCA pump can transform a transient pERK1/2 signal into a plateau signal with no decay over time (4B, blue). Increasing the amplitude of the CRAC channel (with baseline fitted value of 1.74x10^4^ μM/s) is also predicted to have a significant effect on the duration of the pERK1/2 signal as demonstrated in Fig 4C. Sufficient increase in CRAC channel current amplitude can lead to a sustained pERK1/2 signal with no decay (Fig 4C, pink and green). We next consider the concentration of the calcium binding protein CIB1 that is critical for SphK1 translocation to the plasma membrane in the model. The concentration of CIB1 is varied and the effects on pERK1/2 are considered. ERK1/2 activation is insensitive to total concentration of CIB1 higher than 0.5 μM (baseline value is 0.5 μM). However, pERK1/2 monotonically decreases as the CIB1 is reduced from 0.5 μM to 0 (Fig 4D). Sample pERK1/2 versus time curves in response to variations in CIB1 concentration are shown in Fig 4E. For sufficiently small values of CIB1 concentrations (5nM), there is no ERK1/2 activation (Fig 4E, blue). We next considered the effect on pERK1/2 of altering the dissociation constant for the binding of CIB1 to SphK1. As shown in Fig 4F, the changes in the binding constant affect maximum pERK1/2. Sample traces are shown in Fig 4G. The change in binding affects both the duration and the amplitude of the pERK1/2 signal. Another important parameter in SphK1 activation is CIB1/SphK1 translocation rate constant from the cytoplasm to the plasma membrane. The maximum pERK1/2 is relatively stable for translocation rates down to 0.1 s^-1^ (Fig 4H). However, below this value, the maximum pERK1/2 rapidly and monotonically declines to zero. In fact, a lower bound of 0.044 s^-1^ (time constant of ~22 s) is predicted to be necessary for ERK1/2 activation. Sample traces are also shown in Fig 5I. The translocation time course of CIB1/SphK1 is thus predicted to be an important regulator of the pERK1/2 dynamics in response to VEGF.

Overall, these simulations predict that pERK1/2 shape and dynamics can be strongly modulated by the perturbations in calcium dynamics including the PMCA rate, SERCA pump rate, and the current through the CRAC channels. ERK1/2 activation is also regulated by the concentration of CIB1 protein, the strength of CIB1 binding to SphK1, and the translocation time constant of the activated CIB1 from the cytoplasm to the plasma membrane.

We also carried out simulations to evaluate the effect of receptor-level perturbations on ERK1/2 activation. These included changes in VEGFR2 internalization rate (S3 Fig), dephosphorylation rate (S4 Fig), and the number of receptors (S5 Fig). The main result from these simulations is that ERK1/2 activation is predicted be stable in response to a wide range of changes in parameters that determine receptor dynamics.

### Dependence of ERK1/2 activation on phosphorylated VEGFR2 signal and implications for therapeutic interventions targeting VEGF signaling to ERK1/2

Considering signal transduction from VEGFR2 to downstream species, it is tempting to assume that ERK1/2 activation is linearly correlated with the extent of phosphorylated VEGFR2. Our hypothesis was that the effect of SphK1 feedback on ERK1/2 activation would result in a fundamentally different relation between pERK1/2 and pVEGFR2. In fact, we already observed that the model predicted the existence of threshold behavior in ERK1/2 activation in response to VEGF concentration. In this section, we investigate the behavior of pERK1/2 versus pVEGFR2 predicted by the model. As shown in Fig 5A, pERK1/2 is relatively insensitive to changes in the phosphorylation rate down to 98.5% of the baseline phosphorylation rate. Further, pERK1/2 exhibits a threshold behavior in response to receptor phosphorylation. For values larger than 0.64 s^-1^ (1.5% of the control value), ERK1/2 activation is unscathed. Plotting maximum pERK1/2 versus maximum fractional pVEGFR2 (fraction of total VEGFR2) results in a highly non-linear relation presented in Fig 5B.

The model predicts that there is a threshold fraction of pVEGFR2 below which there is no ERK1/2 activation. This threshold value is ~4.6%, meaning that only 4.6% of the total receptors need be phosphorylated to induce a robust ERK1/2 activation. SphK1-dependent ERK1/2 activation is therefore robust to variations in phosphorylated VEGFR2 population, implying that above 4.6% fractional pVEGFR2, ERK1/2 activation is not fundamentally altered by changes in pVEGFR2 fraction. For instance, reducing pVEGFR2 fraction from 30% to 10%, does not significantly affect ERK1/2 activation and the sole predicted effect is a reduction in maximum pERK1/2 from ~0.23 to ~0.15 (Fig 5B). We next investigated parameters within the SphK1 feedback that might alter this threshold value. As shown in Fig 5C, pVEGFR2 threshold value in ERK1/2 activation is strongly modulated by the concentration of SphK1 in the cell. 50% reduction in SphK1 concentration (baseline value of 0.1 μM), increased the threshold value from 4.6% to ~9%. Increasing SphK1 to 1 μM, reduced the threshold further to 0.8%, implying that under conditions where SphK1 is overexpressed in the cell, less than 1% of the receptors need be phosphorylated for robust ERK1/2 activation. Similar pattern held true for kdp_S1P_, a parameter determining the dephosphorylation rate of S1P (Fig 5D). Increasing S1P dephosphorylation by 4-fold, increased the threshold value from 4.6% to 15%. Decreasing S1P 10-fold, reduced the threshold value from 4.6% to 0.8%, once again implying that under heightened S1P levels, less than 1% of the receptors are required to be phosphorylated for effective ERK1/2 activation. Threshold behavior is also predicted for the active SphK1 (Fig 5E) and S1P (Fig 5F), implying the existence of intimate connection between SphK1 and ERK1/2 dynamics. The model thus proposes a radical hypothesis that SphK1 feedback endows the cells with the ability to effectively activate ERK1/2 in response to small fraction of active VEGFR2 on the cell and effectively shield downstream ERK1/2 activation from slight perturbations in receptor phosphorylation rate. The model was then utilized to simulate the anti-angiogenic strategies targeting VEGF pathway. Fig 5G simulates the effect of depleting VEGF (with an agent such as bevacizumab). Model demonstrates that VEGF depletion alone is ineffective in blocking ERK1/2 activation. The figure shows that over 99% of the external VEGF need be depleted before any inhibitory effect on pERK1/2 is observed. Similarly, Fig 5H shows the result of inhibiting the phosphorylation rate with a generic tyrosine kinase inhibitor (TKI) such as sunitinib that primarily inhibits receptor phosphorylation. Once again over 99% inhibition is necessary. The model thus predicts that TKIs and antibodies that solely target VEGFR2 autophosphorylation and VEGF are not effective in shutting down signaling to ERK1/2. Depleting of VEGFR2 by an anti-VEGFR2 mAb agent, e.g., ramucirumab is predicted to have a lower threshold of inhibition at 66%, but ERK1/2 activation is robust until the threshold depletion level is achieved (Fig 5I). This level of inhibition might still be very challenging to achieve in an *in vivo* setting where antibody delivery to tumors is a confounding effect. Moreover, ERK1/2 activation is robust up to 66% VEGFR2 depletion. Next we simulate a combined inhibition of receptor phosphorylation and Raf by a generic small molecule TKI such as sorafenib (Fig 5J). This is much more effective in gradually inhibiting pERK1/2 amplitude until complete inhibition at 78%. This is in contrast with previous cases where drop in ERK1/2 amplitude to zero is very sudden (switch-like) occurring at a threshold inhibition level. Combining sorafenib and ramucirumab is even more effective achieving full blockade of pERK1/2 at 41% inhibition (Fig 5K). Combining sorafenib and ramucirumab with an anti-SphK1 agent further improves the inhibitory potential reducing the threshold for inhibition to 36%. These simulations demonstrate that combination therapy is essential in achieving efficient and sustained (pERK1/2 amplitude decreasing as a function of percent inhibition as in Fig 5J, 5K and 5L) inhibition of VEGF signaling to ERK1/2.

## Discussion

### VEGF threshold behavior in ERK1/2 activation

An important prediction of the model is the existence of threshold in VEGF activation of ERK1/2. This implies that there is a critical value of VEGF concentration below which ERK1/2 activation ceases. The EC50 for the activation of VEGFR2 by VEGF is ~30 pM [43]. Soluble VEGF is capable of activating ERK1/2 in concentrations as low as ~12 pM in HUVEC [44] and ~6 pM in PAEC [45]. What emerges from Constraining the model with these experimental data is the existence of VEGF threshold of ~5 pM for ERK1/2 activation. An interesting observation in [44] was that in the absence of the heparin binding domain of fibronectin, the antibody to phosphorylated VEGFR2 did not detect a signal at 1 ng/ml (24 pM), while pERK1/2 signal was still detectable down to VEGF concentrations of 0.5 ng/ml (12pM). One experimentally verifiable prediction of the model is that by overexpressing SphK1, one should be able to increase the lower bound for the activation of soluble VEGF. The pERK1/2 versus pVEGFR2 curve (Fig 5B) is also revealing, demonstrating that robust activation of ERK1/2 is achieved with only 4.6% of the receptors phosphorylated. The small population of phosphorylated receptors required for activation of ERK1/2, might be the reason for the difficulty in detecting phosphorylated receptors at low VEGF concentrations while being able to detect the pERK1/2 signal. The model provides a rationale and motivation for performing further experiments at more physiological concentrations of VEGF, even in the absence of detectable phosphorylated receptor species.

### Calcium modulation of ERK1/2 activation

Calcium elevation is essential for angiogenic response to VEGF, and inhibition of VEGF-mediated calcium influx prevents endothelial cell proliferation [48,52,53]. Moreover, strong buffering of cytoplasmic calcium blocks ERK1/2 activation downstream of VEGF [54]. To our knowledge, the computational model developed here is the first of its kinds to incorporate specific calcium cycling mechanisms downstream of VEGFR2, including the CRAC channels that have been experimentally shown to be critical for angiogenesis [36]. The calcium influx through the CRAC channels is predicted to alter the plateau phase and duration of the pERK1/2 signal. Indeed, complete inhibition of CRAC channels abolishes the pERK1/2 plateau phase.

Experimentally, calcium channel inhibition has been explored as a viable target in cancer for inhibiting angiogenesis in solid tumors [55] and ovarian cancer [56]. Moreover, some types of cancer have been shown to down-regulate the expression PMCA and SERCA pumps [57] that are predicted by the model to increase the duration and amplitude of pERK1/2 signal (Fig 4A and 4B). The model predicts that calcium signaling should not be overlooked when investigating the activation of angiogenic pathways by VEGF. Further experimental evidence is needed to elucidate and test the predicted link between calcium dynamics and ERK1/2 activation.

Overall, the model predicts that changes in the amplitude and duration of the calcium transient by interventions such as changes in the activity of SERCA and PMCA pumps, and CRAC channels may modulate VEGF-dependent ERK1/2 signaling. Moreover, any therapeutic agent that interferes with calcium signaling might also have far-reaching effects on VEGF-mediated angiogenesis.

### Implications for anti-angiogenic therapy in cancer

In cancer, the goal is to inhibit angiogenesis and prevent tumor vascularization and growth. Our simulations show that antibodies such as bevacizumab [58,59] that target VEGF would not be effective at shutting down VEGF signaling to ERK1/2 unless 99% inhibition of VEGF is achieved. This high threshold for ERK1/2 inhibition would seriously hinder the applicability of the antibody as an effective anti-angiogenic agent and might explain the limited increase in overall survival (usually less than 6 months) in cancers responding to bevacizumab treatment such as metastatic colorectal cancers [60–62], non-small-cell lung cancer (NSCLC) [63,64], metastatic renal cell carcinoma [65], and ovarian cancer [66]. In metastatic breast cancer, bevacizumab resulted in no improvements in overall survival [67]. While our model has focused on one specific pathway (namely ERK1/2), it does show that even within the context of a single pathway, monotherapy in the form of anti-VEGF antibody would not be effective unless the concentrations are sufficiently high so that 99% of the ligand molecules are sequestered. This is a very stringent requirement in any *in vivo* setting, especially that we are not even including the very real possibility of developing resistance to anti-VEGF therapies [68]. Similarly, simulating the inhibition of VEGFR2 autophosphorylation by a generic TKI such as sunitinib that primarily inhibits receptor phosphorylation indicates that over 99% inhibition is necessary before signaling to ERK1/2 is compromised. Sunitinib has been approved for use in advanced renal cell carcinoma and increases median overall survival from 21.8 to 26.4 months [69]. What is suggested by our modeling exercise is that the high threshold for inhibition of VEGF-ERK1/2 signaling might explain some of the difficulties in effective inhibition of VEGF-mediated angiogenesis with TKIs in various types of cancers. The model also emphasizes the absolute necessity of developing more efficient TKI drug delivery strategies to enhance local concentration of the drug in tumor microenvironment in order to overcome the inhibition threshold.

An interesting prediction from the model is that compared to other mono-therapies (agents targeting a single node of the VEGF pathway), depleting VEGFR2 with an antibody such as ramucirumab should be more effective, exhibiting lower inhibition threshold (~66%). Once again, ERK1/2 activation is robust up until the threshold level of inhibition is reached. Similar to other agents, ramucirumab has shown limited efficacy in certain types of tumors such as advanced gastric [70] and metastatic advanced non-small-cell lung carcinoma [71].

Our simulations suggests that when it comes to inhibiting VEGF signaling to downstream effectors such as ERK1/2, combination therapy seems to be essential. In fact, according to the model, TKIs such as sorafenib are more effective because they inhibit signaling at both the receptor level (VEGFR2 phosphorylation) and a downstream effector node (Raf). It is indeed interesting to note that sorafenib is the only FDA approved anti-angiogenic agent for hepatocellular carcinoma (HCC) and the only TKI (in the list of inhibitors in clinical trials for HCC) that targets two distinct nodes of the VEGF/VEGFR2 pathway [72].

Simulations demonstrate more effective combination strategies. For example, combining ramucirumab and sorafenib achieves a lower threshold of inhibition (41% according to the model) with rapid decline in maximum pERK1/2 as a function of inhibition. Another example is triple combination involving sorafenib, ramucirumab, and an inhibitor for Sphk1 pathway (such as sphingomab [73]) that is predicted to further improve the inhibitory effects on VEGF-ERK1/2 axis.

In all, the model developed here demonstrates some of the challenges in developing effective anti-angiogenic therapies targeting the VEGF pathway and highlights the need to consider specific pathway dynamics (e.g. threshold behavior) and structure (e.g. positive and negative feedback loops) when evaluating therapeutic interventions. The most clinically relevant prediction from the current model is that even in inhibiting a single pathway involving VEGF signaling to ERK1/2 and in the absence of any consideration of tumor resistance, combination therapeutic strategies seem to be essential.

### Model limitations and future extension

There are several aspects of the model that can be improved in the future. The model includes only a single phosphorylation site on VEGFR2. The modular structure of BioNetGen allows for additional phosphorylation sites to be included and investigated. These can in principle be readily included and investigated in the future. Another limitation is the simplified description of receptor recycling that includes only two compartments, namely the surface receptors and the receptors within the signaling endosomes. Including Rab specific compartments similar to the model in [26] would significantly increase model complexity and molecular detail.

The calcium cycling module includes a phenomenological description of the current through CRAC channels as a function of calcium concentration within the ER lumen. This model can be improved by including dynamic STIM oligomerization similar to the model in [74]. The model also does not include TRPC calcium channels that are regulated by DAG and are shown to be important in VEGF-mediated angiogenesis [52,75]. As additional data with specific inhibitors of TRPC channels become available, the relation between TRPC signaling and ERK1/2 activation downstream of VEGF would be a fruitful avenue of investigation in the model.

Pertaining to SphK1 signaling, a confounding pathway is the activation of S1P receptors [76,77] (S1PR1 and S1PR2) by the S1P generated downstream of VEGF. Including this receptor would go well beyond the scope of the current study; however, rule-based modeling would indeed be very suitable for studying the interaction between VEGFR2 and S1PRs at the receptor and downstream levels and the future versions of the model can include this important pathway.

## Methods

The rules for the interaction of the receptors and downstream signaling details are incorporated into the BioNetGen text file and can be accessed with ease and is included in the supplementary material. We have also supplemented the SBML file associated with the model. The binding of VEGF to VEGFR1, VEGFR2, and NRP1 follows standard kinetic schemes similar to previous studies [32,78]. List of parameters with their descriptions is also includes in S1 Table. S2 Table contains the initial values for the seed species in the model. The rules generate 208 species and 932 reactions. The binding of PLCγ to pVEGFR2 and subsequent phosphorylation and dissociation of PLCγ from the receptor is described by a Michaelis-Menten type reaction as follows:

$$\begin{array}{l} \text{pVEGFR}2_{surface}\;+\text{ PLC}\gamma \;\to \text{ pVEGFR}2_{surface}\;+\text{ pPLC}\gamma \\ \text{Rate}=\frac{kpPLC\gamma [pVEGFR2_{surface}][PLC\gamma ]}{[PLC\gamma ]+Km_{PLC\gamma /R2}} \end{array}$$ (1)

$$\begin{array}{l} \text{pVEGFR}2_{membrane}\;+\text{ PLC}\gamma \;\to \text{ pVEGFR}2_{membrane}\;+\text{ pPLC}\gamma \\ \text{Rate}=\frac{kpPLC\gamma [pVEGFR2_{membrane}][PLC\gamma ]}{[PLC\gamma ]+Km_{PLC\gamma /R2}} \end{array}$$ (2)

Note that BioNetGen accounts for all the VEGFR2 species that are phosphorylated. This approach significantly lowers the number of reactions generated by the rules and prevents combinatorial explosion in the model. Another aspect of the model is the generation of active Ras (RasGTP) by S1P. As the precise link between S1P and Ras activation is not entirely clear, we assume a Michaelis-Menten type reaction:

$$\begin{array}{l} \text{RasGDP}+\text{S}1\text{P}\to \text{RasGTP}+\text{S}1\text{P}\\ \text{Rate}=\frac{k_{\text{S}1\text{P}/\text{Ras}}\left[\text{S}1\text{P}\right]}{\left[\text{S}1\text{P}\right]+km_{\text{S}1\text{P}/\text{Ras}}} \end{array}$$ (3)

with parameters k_S1PRas_ and K_m,S1PRas_ determining the strength of Ras activation by S1P. More details of calcium cycling and SphK1 activation module are given in the supplementary material.

BioNetGen created the network as a set of reactions and the corresponding ordinary differential equations (ODEs) saved as a C file, readable with MEX functionality in MATLAB (Mathworks, 2015). The set of ODES was numerically integrated using SUNDIAL numerical solver suite [79]. For parameter fitting, we applied a direct search algorithm implemented in the MATLAB function *patternsearch* as part of the global optimization toolbox. All the pieces of data, including the surface and total receptor levels and downstream activation were fitted simultaneously in MATLAB.

Data from the western blot images were extracted using the software imageJ [80].

Global sensitivity analysis was performed using the partial rank correlation coefficient (PRCC) algorithm described in [51]. The parameter values were randomly chosen from a uniform distribution within a range 0.01 × fitted_value ≤ *p* ≤ 20 × fitted_value.

## Supporting information

S1 Fig Predicted activation dynamics of PKC, Raf, and Ras downstream of VEGFR2. **A**. The predicted active PKC in response to 50 ng/ml VEGF, **B**. RasGTP transient predicted by the model, **C**. pRaf transient in response to 50 ng/ml VEGF, **D**. Active Ras versus VEGF dose-response exhibiting threshold behavior, with the threshold value of VEGF = 5pM,**E**. Cytosolic calcium concentration in response to different values of CRAC channel amplitude, **F**. Cytosolic calcium concentration in response to IP3R current amplitude.
(TIFF)

S2 Fig Raf concentration and VEGF threshold for ERK1/2 activation. **A**. pERK1/2 dose response relative to VEGF, computed in response to different Raf concentrations, **B**. VEGF threshold relative to Raf shows a negative monotonic response. Increase in Raf, decreases the threshold value of VEGF, **C**. Sample traces of pERK1/2 versus time, computed for different values of Raf concentrations, indicating a strong dependence of the pERK1/2 signal on Raf.
(TIFF)

S3 Fig VEGFR2 activation and downstream signaling as a function of receptor internalization rate. **A**. The amplitude of pVEGFR2 as a function of receptor internalization rate, **B**. Max intracellular calcium in response to variations in internalization rate, **C**. Max pERK1/2 versus the rate of receptor endocytosis showing the existence of threshold at 2.5% of the control value, **D**. pVEGFR2 versus time traces for four different internalization rates, **E**. Intracellular calcium traces for various internalization rates, **F**. pERK1/2 versus time traces for four different values of receptor endocytosis.
(TIFF)

S4 Fig VEGFR2 activation and downstream signaling as a function of receptor dephosphorylation rate in endosomal compartment. **A**. The amplitude of pVEGFR2 as a function of dephosphorylation rate of the internalized receptors, **B**. Max intracellular calcium in response to variations in internalized receptor dephosphprylation rate, **C**. Max pERK1/2 versus the rate of receptor dephosphorylation showing the existence of a threshold above which no ERK1/2 activation occurs, **D**. pVEGFR2 versus time traces for four different dephosphorylation rates, **E**. Intracellular calcium traces, **F**. pERK1/2 versus time.
(TIFF)

S5 Fig Response of pERK1/2 to receptor level perturbations. **A**. Maximum value of pERK1/2 relative to changes in number of VEGFR2 (R2). The maximum value of pERK1/2 decreases monotonically as R2 is decreased until receptor numbers reach a critical value of 1.6 #/μm^2^. **B**. Sample traces for pERK1/2 versus time curves for different receptor numbers (multiples of baseline receptor number), **C**. Maximum fractional value of pERK1/2 (blue) versus the number of VEGFR1 (R1) showing that the value decreases from 0.25 to 0.15, **D**. pERK1/2 versus time curves for different VEGFR1 numbers, **E**. The changes in maximum pERK1/2 relative to NRP1 numbers, **F**. Sample pERK1/2 versus time curves for different NRP1 numbers,**G**. The effect of the dissociation constant for the binding of VEGF to NRP1 on maximum pERK1/2, **H**. pERK1/2 versus time for different values of kd_VEGF/NRP1_.
(TIFF)

S1 Table Model parameters. (PDF)

S2 Table Initial values for the seed species in the model. (PDF)

S1 Model Description Extended description of the model. (PDF)

S1 SBML File Systems biology markup language (SBML) file for the model. (XML)

S1 BioNetGen Text Text of the BioNetGen file. (DOCX)

S1 Species List List of the species generated by the model. (XLSX)

S1 Reaction List List of reactions generated and simulated by the model. (XLSX)
