# Supplementary figures and images for: Computational investigation of sphingosine kinase 1 (SphK1) and calcium dependent ERK1/2 activation downstream of VEGFR2 in endothelial cells
Source: PLoS Comput Biol. 2017 Feb 8;13(2):e1005332. doi: 10.1371/journal.pcbi.1005332 (PMC5298229; doi:10.1371/journal.pcbi.1005332)

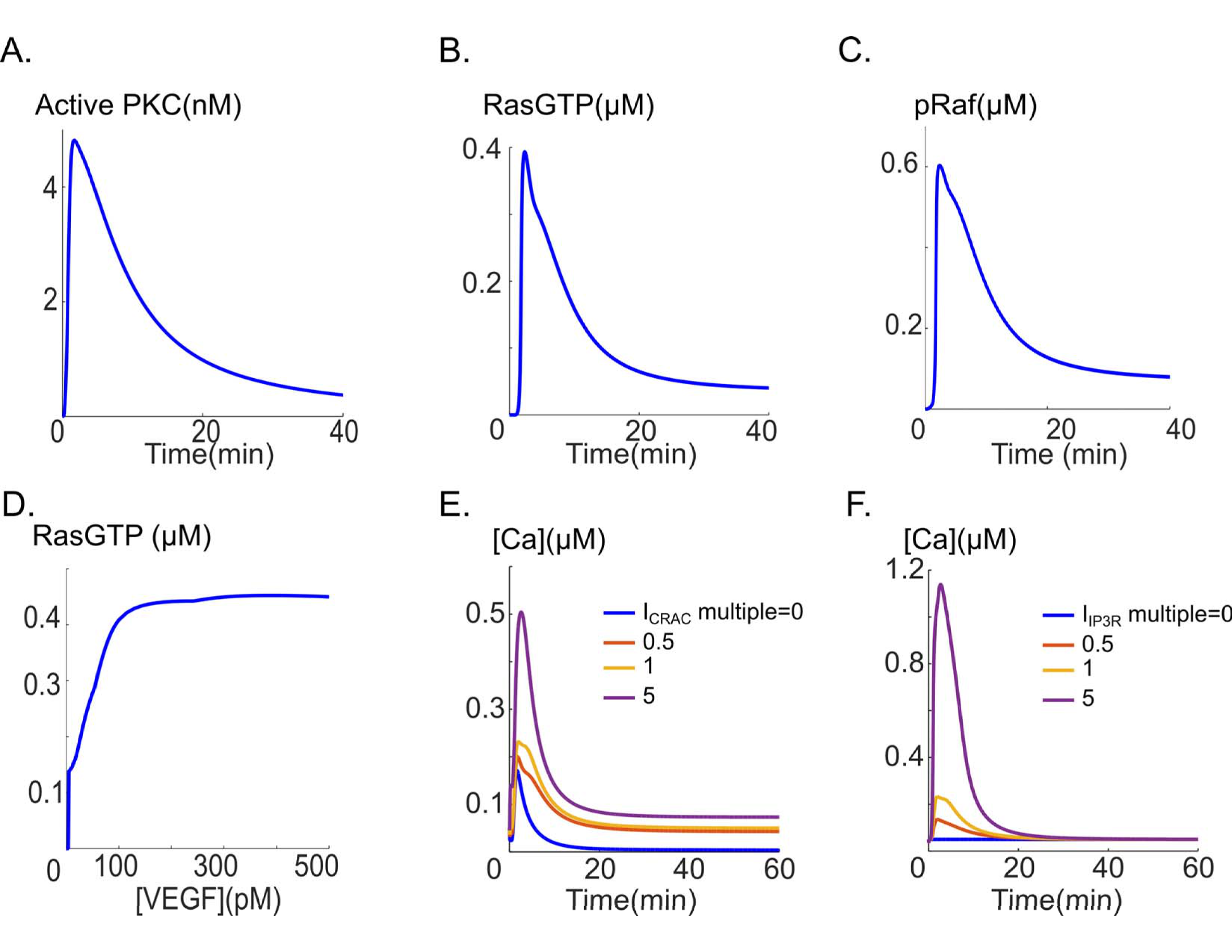

Supplement: S1 Fig — A. The predicted active PKC in response to 50 ng/ml VEGF, B. RasGTP transient predicted by the model, C. pRaf transient in response to 50 ng/ml VEGF, D. Active Ras versus VEGF dose-response exhibiting threshold behavior, with the threshold value of VEGF = 5pM,E. Cytosolic calcium concentration in response to different values of CRAC channel amplitude, F. Cytosolic calcium concentration in response to IP3R current amplitude. (TIFF) [file pcbi.1005332.s001.tiff]

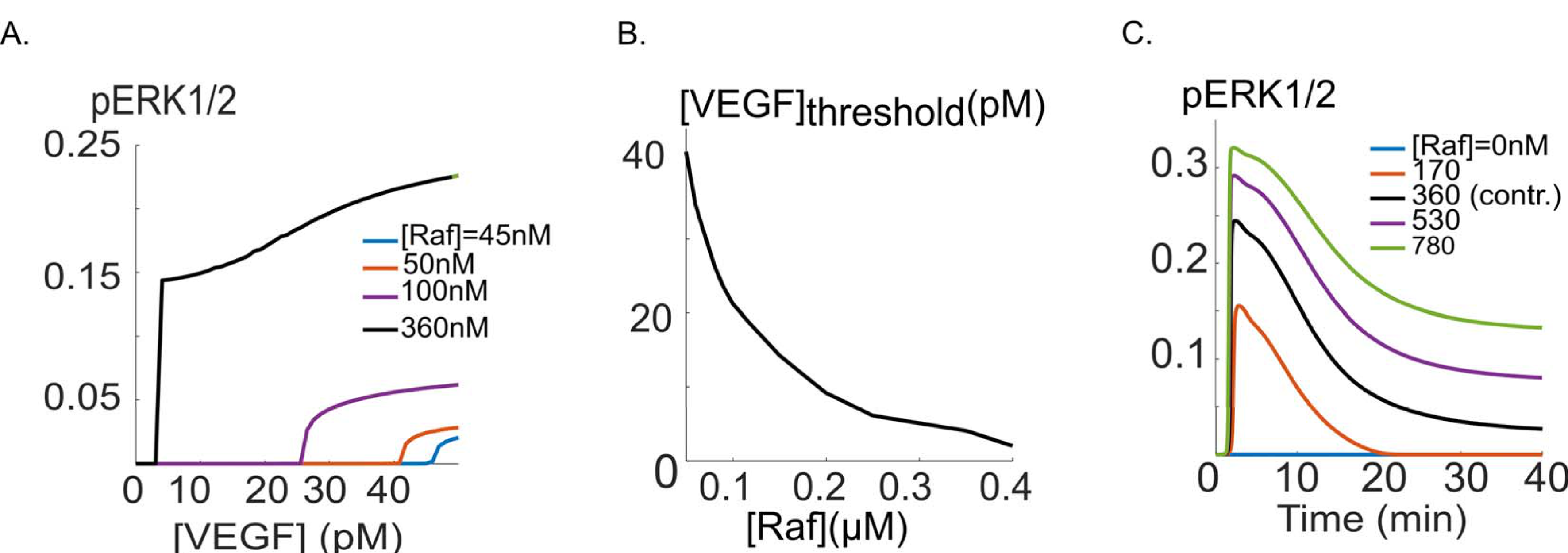

Supplement: S2 Fig — A. pERK1/2 dose response relative to VEGF, computed in response to different Raf concentrations, B. VEGF threshold relative to Raf shows a negative monotonic response. Increase in Raf, decreases the threshold value of VEGF, C. Sample traces of pERK1/2 versus time, computed for different values of Raf concentrations, indicating a strong dependence of the pERK1/2 signal on Raf. (TIFF) [file pcbi.1005332.s002.tiff]

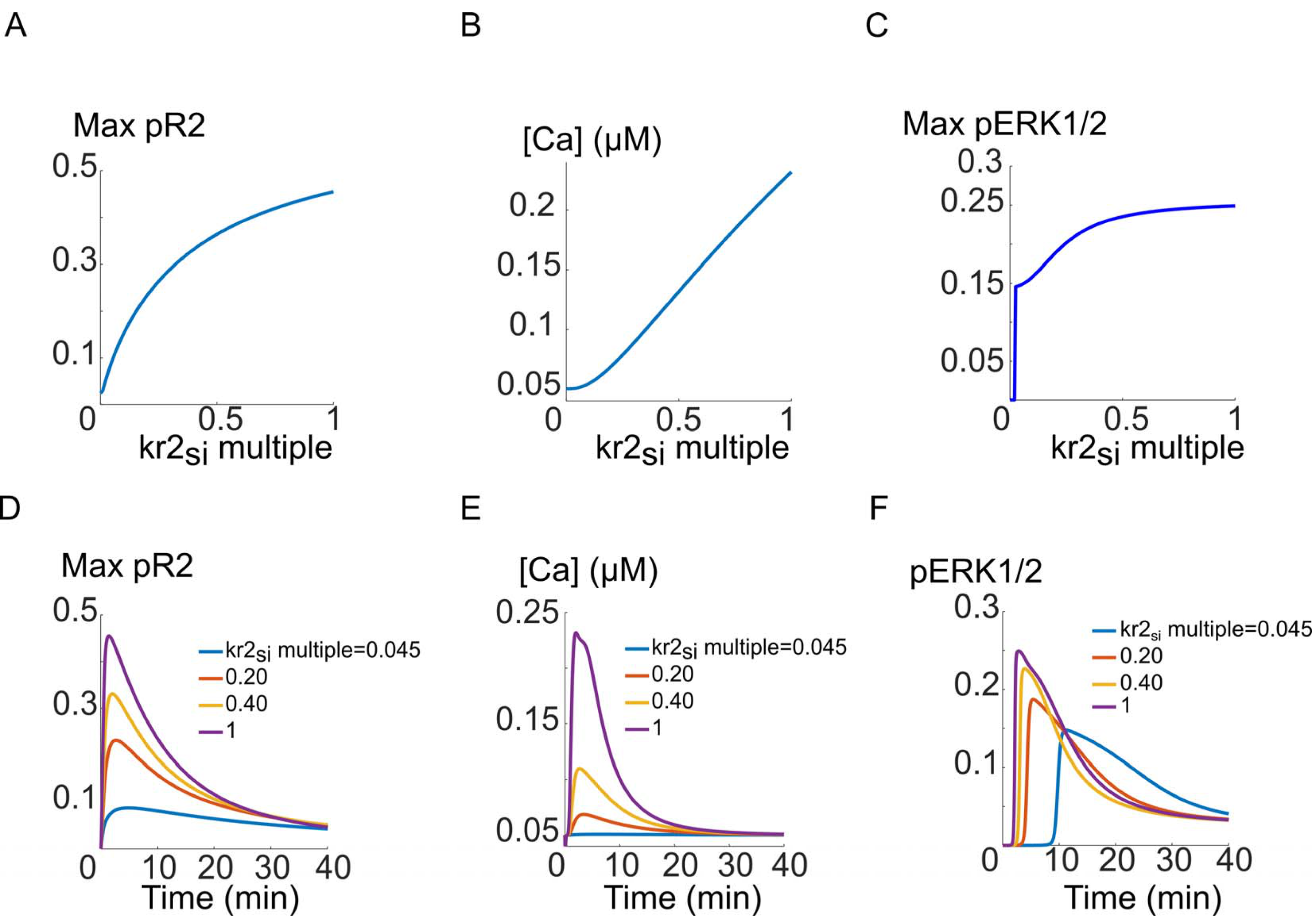

Supplement: S3 Fig — A. The amplitude of pVEGFR2 as a function of receptor internalization rate, B. Max intracellular calcium in response to variations in internalization rate, C. Max pERK1/2 versus the rate of receptor endocytosis showing the existence of threshold at 2.5% of the control value, D. pVEGFR2 versus time traces for four different internalization rates, E. Intracellular calcium traces for various internalization rates, F. pERK1/2 versus time traces for four different values of receptor endocytosis. (TIFF) [file pcbi.1005332.s003.tiff]

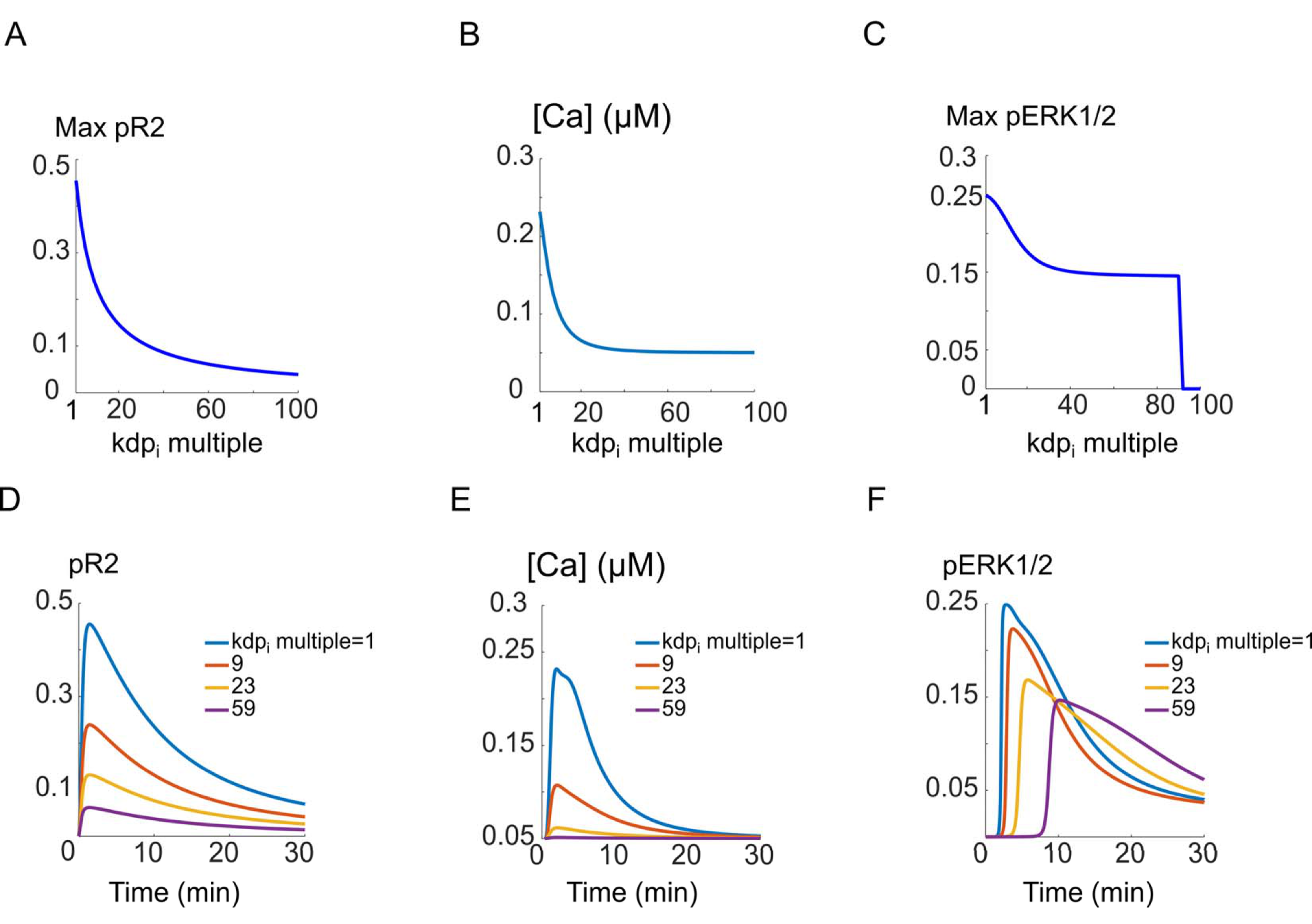

Supplement: S4 Fig — A. The amplitude of pVEGFR2 as a function of dephosphorylation rate of the internalized receptors, B. Max intracellular calcium in response to variations in internalized receptor dephosphprylation rate, C. Max pERK1/2 versus the rate of receptor dephosphorylation showing the existence of a threshold above which no ERK1/2 activation occurs, D. pVEGFR2 versus time traces for four different dephosphorylation rates, E. Intracellular calcium traces, F. pERK1/2 versus time. (TIFF) [file pcbi.1005332.s004.tiff]

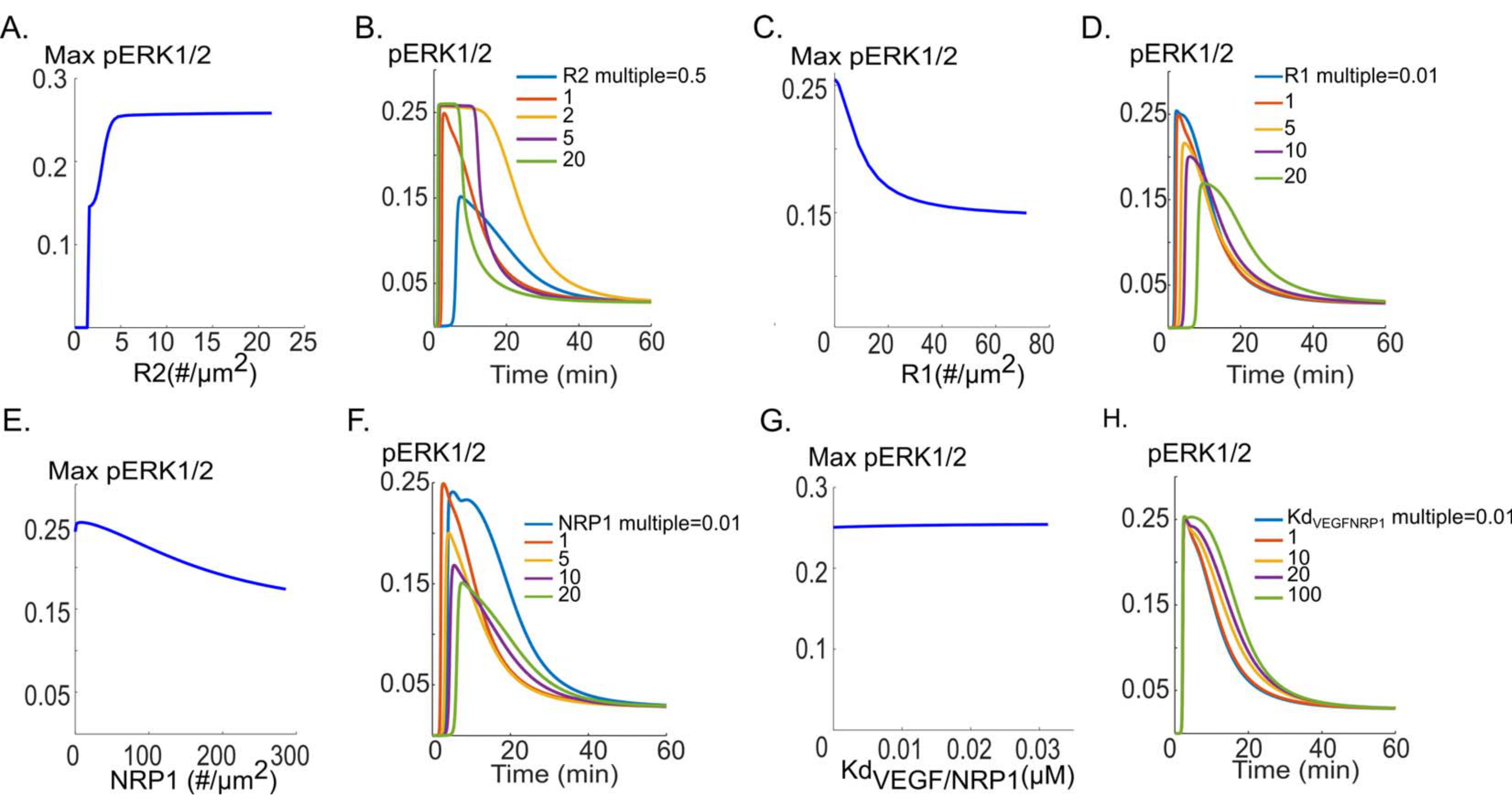

Supplement: S5 Fig — A. Maximum value of pERK1/2 relative to changes in number of VEGFR2 (R2). The maximum value of pERK1/2 decreases monotonically as R2 is decreased until receptor numbers reach a critical value of 1.6 #/μm2. B. Sample traces for pERK1/2 versus time curves for different receptor numbers (multiples of baseline receptor number), C. Maximum fractional value of pERK1/2 (blue) versus the number of VEGFR1 (R1) showing that the value decreases from 0.25 to 0.15, D. pERK1/2 versus time curves for different VEGFR1 numbers, E. The changes in maximum pERK1/2 relative to NRP1 numbers, F. Sample pERK1/2 versus time curves for different NRP1 numbers,G. The effect of the dissociation constant for the binding of VEGF to NRP1 on maximum pERK1/2, H. pERK1/2 versus time for different values of kdVEGF/NRP1. (TIFF) [file pcbi.1005332.s005.tiff]
