# Supplementary material for: Computational investigation of sphingosine kinase 1 (SphK1) and calcium dependent ERK1/2 activation downstream of VEGFR2 in endothelial cells
Source: PLoS Comput Biol. 2017 Feb 8;13(2):e1005332. doi: 10.1371/journal.pcbi.1005332 (PMC5298229; doi:10.1371/journal.pcbi.1005332)
Supplement: S1 Table — (PDF) [file pcbi.1005332.s006.pdf]

Table S1. Model parameters

Name	Description	Value (units)	Reference
Vol_{cyto}	Cytoplasmic volume	9.12×10^{-13} L	[1]
Vol_{ext}	External volume	2×10^{-3} L	Solution volume in the dish
Vol_{ER}	ER volume	3.35×10^{-13} L	[1]
A_{cell}	Cell area	1400 μm^2	[2]
$VEGF165_0$	VEGF165A concentration	$1.19\times10^{-3}\mu M$ (50 ng/ml)	standard
$VEGFR2_0$	Total VEGFR2	4.29 $\#/\mu m^2$ (6000 receptors)	[3]
$VEGFR1_0$	Total VEGFR1	1.43 $\#/\mu m^2$ (2000 receptors)	[3]
$NRP1_0$	Total NRP1	28.6 $\#/\mu m^2$(40000 receptors)	[4]
kvr_{on}	The on kinetics of binding of VEGF to VEGFR2	4.4 $\mu M^{-1}s^{-1}$	[5]
kvr_{off}	Thee off kinetics of binding of VEGF to VEGFR2	2.6×10^{-2} s^{-1}	[5]
kc_{VR}	The on kinetics of binding of VEGF-VEGFRx species to VEGFRx receptors (x=1 or 2)	2.13×10^{-3} $\#^{-1}\mu m^2 s^{-1}$	Fit
kc_{RR}	Ligand-independent coupling of the receptors on kinetics	1.11 $\#^{-1}\mu m^2 s^{-1}$	Fit
kd_{RR}	Ligand-independent coupling of the receptors off kinetics	0.78 s^{-1}	Fit

$kvr1_{on}$	The on kinetics of binding of VEGF to VEGFR1	22 $\mu M^{-1}s^{-1}$	[5]
$kvr1_{off}$	Thee off kinetics of binding of VEGF to VEGFR1	2.6×10^{-2} s^{-1}	[5]
$k\Delta_{RR}$	The on kinetics of coupling of receptors within a hetero – or homo- dimer.	2.50 s^{-1}	[5] and Fit
$k\Delta_{VR}$	The on kinetics of binding of a receptor to VEGF within a hetero – or homo- dimer.	2.05s^{-1}	[5] and Fit
kp_{Y1175}	Phosphorylation rate of Y1175	42.39 s^{-1}	Fit
kdp_{s}	De-phosphorylation rate of Y1175 at the membrane	7.69×10^{2} s^{-1}	Fit
kdp_{i}	De-phosphorylation rate of Y1175 for internalized receptors	5.44 s^{-1}	Fit
$kr2_{si}$	Rate of internalization of ligand-bound VEGFR2 without NRP1 in the complex	6.1×10^{-2} s^{-1}	Fit
$kr2_{is}$	Endosome to membrane shuttling rate	$1.24\times10^{-3}s^{-1}$	Fit
$ksingleR2_{si}$	Internalization rate o VEGFR2 in the absence of ligand	9.23×10^{-4} s^{-1}	Fit
$ksingleR2_{is}$	Endosome to membrane shuttling rate of VEGFR2 in the absence of ligand	0.267 s^{-1}	Fit

$kdeg_{i0}$	Degradation rate of the internalized phosphorylated VEGFR2	$1.41\times10^{-3}\ s^{-1}$	Fit
$kdeg_{r2/NRP1i0}$	Degradation rate of the internalized phosphorylated VEGFR2 that are bound to at least one NRP1 co-receptor	$1.18\times10^{-2}s^{-1}$	Fit
$kdeg_{i0/notPhos}$	The degradation rate of un-phosphorylated VEGFR2 receptors that are not bound to NRP1 co-receptors	$9.37\times10^{-4}\ s^{-1}$	Fit
$kdeg_{r2/NRP1i0/notPhos}$	The degradation rate of unphosphorylated VEGFR2 receptors that are bound to at least one NRP1 co-receptor	$0.01\ s^{-1}$	Fit
$kr2NRP1_{si}$	Internalization rate of VEGFR2 with NRP1 in the complex	$0.404\ s^{-1}$	Fit
$kr2NRP1_{is}$	Endosome to membrane shuttling rate with NRP1in the complex	$0.756\ s^{-1}$	Fit
$kVEGFNRP1_{on}$	The on kinetics of binding of VEGF to NRP1	$3.2\ \mu M^{-1}s^{-1}$	[6]
$kVEGFNRP1_{off}$	The off kinetics of binding of VEGF to NRP1	$0.001s^{-1}$	[6]
$kNRP1VEGFR2_{on}$	The on kinetics of VEGF.NRP1 binding to VEGFR2	$0.554\ \#^{-1}\mu m^{2}s^{-1}$	Fit

$kNRP1VEGFR2_{off}$	The off kinetics of VEGF.NRP1 binding to VEGFR2	4.891 s^{-1}	Fit
$kNRP1VEGFR1_{on}$	The on kinetics of NRP1 binding to VEGFR1	1.31 $\#^{-1}\mu m^2 s^{-1}$	Fit
$kNRP1VEGFR1_{off}$	The off kinetics of NRP1 binding to VEGFR1	0.1 s^{-1}	Fit
$kVEGFR2NRP1_{on}$	The on kinetics of binding of VEGF.VEGFR2 to NRP1	5.7×10^{-3} $\#^{-1}\mu m^2 s^{-1}$	Fit
$kVEGFR2NRP1_{off}$	The off kinetics of binding of VEGF.VEGFR2 to NRP1	5.05 s^{-1}	Fit
$kVEGFNRP1_{on}$	The on kinetics of binding of VEGF.VEGFR1 to NRP1	1.56×10^{-2} $\#^{-1}\mu m^2 s^{-1}$	Fit
$kVEGFNRP1_{off}$	The off kinetics of binding of VEGF.VEGFR1 to NRP1	$5.36 s^{-1}$	Fit
$kPIP2_{gen}$	Rate of PIP2 generation	$4.8\times10^{-5} s^{-1}$	[7]
$kSph_{gen}$	Rate of Sphingosine generation	$4.8\times10^{-5} s^{-1}$	Same as PIP2
$kp_{PLC\gamma}$	Phosphorylation rate of PLCγ	0.1 s^{-1}	Fit
$Km_{PLC\gamma/R2}$	Michaelis-Menten type constant for the activation of PLCγ by phospho-VEGFR2	8 μM	FIt
$kdp_{PLC\gamma}$	De-phosphorylation rate of PLCγ	0.1 s^{-1}	Fit
$km_{PIP2PLC\gamma}$	Michaelis-Menten type parameter for PLCγ phosphorylation	0.194 μM	Fit

n_{DAG}	The Hill coefficient for the generation of IP3 and DAG by PLCγ	2.495	Fit
$kcat_{PLC\gamma/DAG}$	The catalytic rate of PLC□	0.1 s^{-1}	Fit
$k_{deg,IP3}$	Degradation rate of IP3	9.22×10^{-2}	Fit
$k_{deg,DAG}$	Degradation rate of DAG	0.109 s^{-1}	Fit
$\bar{I}_{IP3R}$	Amplitude of the IP3R current	3.62×10^{4} s^{-1} (2.34×10^{3} pA/μM)	Fit
$K_{m,IP3R}$	IP3 concentration for half-maximal activation of IP3R	1.6μM	[1,8,9]
I_{PMCA}	Maximum plasma membrane calcium pump current	5.98 μMs^{-1}(1.06 pA)	Fit
$K_{m,PMCA}$	Calcium concentration fohalf maximal plasma membrane pump activity	0.26 μM	[1]
I_{SERCA}	Maximum ER calcium pump current	4.77 μMs^{-1} (0.84pA)	Fit
$Kleak_{ER}$	ER Calcium leak parameter	7.48×10^{-8} $\mu M^{-1}s^{-1}$	Fit
Km_{SERCA}	Calcium concentration for half maximal activity of the ER calcium pump	0.15 μM	[1]
$K_{i,Ca}$	Ca inhibition of IP3R	1 μM	[9]
$K_{a,Ca}$	Ca activation of IP3R	0.1 μM	[10]
$k_{B,on}$	The on kinetics of binding of calcium to cytoplasmic calcium buffers	100 $\mu M^{-1}s^{-1}$	[9]

$k_{B,off}$	The off kinetics of binding of calcium to cytoplasmic calcium buffers	300 s^{-1}	[9]
$kon_{Ca/NCaM1}$	The on rate for the binding of calcium to the first EF-hand domain on the N-lobe of calmodulin	25 $\mu M^{-1}s^{-1}$	[11]
$koff_{Ca/NCaM1}$	The off rate for the binding of calcium to the first EF-hand domain on the N-lobe of calmodulin	1000 s^{-1}	[11]
$kon_{Ca/NCaM2}$	The on rate for the binding of calcium to the second EF-hand domain on the N-lobe of calmodulin	50 $\mu M^{-1}s^{-1}$	[11]
$koff_{Ca/NCaM2}$	The off rate for the binding of calcium to the second EF-hand domain on the N-lobe of calmodulin	500 s^{-1}	[11]
$kon_{Ca/CCaM1}$	The on rate for the binding of calcium to the first EF-hand domain on the C-lobe of CaM	1.2 $\mu M^{-1}s^{-1}$	[11]
$koff_{Ca/CCaM1}$	The off rate for the binding of calcium to the first EF-hand domain on the C-lobe of CaM	10 s^{-1}	[11]

$kon_{Ca/CCaM2}$	The on rate for the binding of calcium to the second EF-hand domain on the C-lobe of CaM	5 $\mu M^{-1}s^{-1}$	[11]
$koff_{Ca/CCaM2}$	The off rate for the binding of calcium to the second EF-hand domain on the C-lobe of CaM	8.5 s^{-1}	[11]
$CSQN_{total}$	Total concentration of calsequesterin (ER calcium buffer)	15000 μM	[12]
Kd_{CSQN}	Dissociation constant for the binding of calcium to CSQN	800 μM	[12]
$kon_{Ca/PKC}$	The on rate for the binding of calcium to PKC	0.3 $\mu M^{-1}s^{-1}$	[13]
$koff_{Ca/PKC}$	The off rate for the binding of calcium to PKC	0.01 s^{-1}	[13]
$kon_{DAG/PKC}$	The on rate of DAG binding to PKC	0.030 $\mu M^{-1}s^{-1}$	Fit
$koff_{DAG/PKC}$	The off rate of DAG binding to PKC	0.124 s^{-1}	Fit
$kon1_{Ca/CIB}$	The on rate of calcium binding to the first EF-hand domain of CIB1	0.0526 $\mu M^{-1}s^{-1}$	Fit (Kd=1.9 μM from [14])
$koff1_{Ca/CIB1}$	The off rate of calcium binding to the first EF-hand domain of CIB1	0.1 s^{-1}	Fit
$kon2_{Ca/CIB1}$	The on rate of calcium binding to the second EF-hand domain of CIB1	0.185 $\mu M^{-1}s^{-1}$	Fit (Kd=0.54 μM from [14])

$koff2_{Ca/CIB1}$	The off rate of calcium binding to the second EF-hand domain of CIB1	0.1 s^{-1}	Fit
$kon_{CIB1/SphK1}$	The on rate for the binding of CIB1 to SphK1	17.603 $\mu M^{-1}s^{-1}$	Fit
$koff_{CIB1/SphK1}$	The off rate for the binding of CIB1 to SphK1	4.403 s^{-1}	Fit
$kcat_{SK1}$	The catalytic rate of SphK1	37.238 s^{-1}	Fit
$Km_{SK1/Sph}$	The Michaelis-Menten type parameter for SphK1 enzymatic activity	0.0294 μM	Fit
kt_{SK1}	Translocation rate of CIB1-bound SphK1 from cytoplasm to the membrane	1 s^{-1}	Fit
$Ktoff_{SK1}$	The off rate of CIB1/SphK1 binding to the plasma membrane	6.67×10^{-4} s^{-1}	Fit
$koff_{SK1}$	The off rate of calcium-free CIB1/SphK1 from the membrane	0.104 s^{-1}	Fit
kdp_{SK1}	Dephosphorylation rate of SphK1	0.0218 s^{-1}	Fit
k_{RasGAP}	The rate of RasGTP hydrolysis by RasGAP	2.941 s^{-1}	Fit
$kon_{Ras/Raf}$	The on rate for the binding of activated Ras to Raf	13.102 $\mu M^{-1}s^{-1}$	Fit
$koff_{Ras/Raf}$	The off rate for the binding of activated Ras to Raf	0.152 s^{-1}	Fit

kp_{Raf}	The rate of autophosphorylation of the activating tyrosine residues on Raf	1.676 s^{-1}	Fit
kdp_{Raf}	The rate of dephosphorylation of Raf for the tyrosine residues	0.895 s^{-1}	Fit
$kdp_{PKC/Raf}$	The rate of dephosphorylation of the PKC-phosphorylated serine residue on Raf	0.720 s^{-1}	Fit
$kp_{MEK12/Raf1}$	The rate of phosphorylation of the first serine residue on MEK1/2 by activated Raf	1.802 s^{-1}	Fit
$kp_{MEK12/Raf2}$	The rate of phosphorylation of the second serine residue on MEK1/2 by activated Raf	1.205 s^{-1}	Fit
$Km_{MEK12/Raf}$	The Michaelis-Menten type parameter for the phosophorylation of MEK1/2 by active Raf	0.807 μM	Fit
kdp_{MEK12_1}	Rate of dephosphorylation of the first serine on MEK1/2	0.112 s^{-1}	Fit
kdp_{MEK12_2}	Rate of dephosphorylation of the second serine on MEK1/2	0.140 s^{-1}	Fit
$kp_{MEK12/ERK12\_1}$	Phosphorylation rate of ERK1 by active MEK1/2	12.149 s^{-1}	Fit
$kp_{MEK12/ERK12\_2}$	Phosphorylation rate of ERK2 by active MEK1/2	0.516 s^{-1}	Fit
kdp_{ERK12_1}	Dephosphorylate rate of ERK1	6.06 s^{-1}	Fit

kdp_{ERK12_2}	Dephosphorylation rate of ERK2	1.053 s^{-1}	Fit
$kcat_{ERK}$	Catalytic rate of active ERK1/2	7.883 s^{-1}	Fit
$Km_{ERK/SK1}$	The Michaelis-Menten type constant for the phosphorylation of SphK1 by active ERK1/2	1.198 μM	Fit
$k_{S1P/Ras}$	Rate constant for the activation of Ras by S1P	1.556 μMs^{-1}	Fit
$Km_{S1P/Ras}$	Concentration of S1P for half-maximal rate of Ras activation	5.899 μM	Fit
kdp_{S1P}	Rate of S1P dephosphorylation	1.188	Fit
$kcat_{PKC}$	The catalytic rate of PKC	10.208 s^{-1}	Fit
$Km_{PKC/Raf}$	The Michaelis-Menten type constant for the phosphorylation of Raf by PKC	0.314 μM	Fit
$\bar{I}_{CRAC}$	The CRAC channel flux amplitude when ER calcium completely depleted (Ca_{ER}=0)	1.74×10^{4} μMs^{-1} (3.06×10^{3} nA)	Fit
K_{CRAC}	ER concentration resulting in half-maximal CRAC current at steady-state	169 μM	[15]
n_{CRAC}	Hill number for the steady-state CRAC channel activation	4.2	[15]
τ_{stim}	The time constant for the activation of the CRAC current	4 s	Fit and [16]

1. Silva HS, Kapela A, Tsoukias NM (2007) A mathematical model of plasma membrane electrophysiology and calcium dynamics in vascular endothelial cells. Am J Physiol Cell Physiol 293: C277-293.
2. Adams DJ, Hill MA (2004) Potassium channels and membrane potential in the modulation of intracellular calcium in vascular endothelial cells. J Cardiovasc Electrophysiol 15: 598-610.
3. Imoukhuede PI, Popel AS (2012) Expression of VEGF receptors on endothelial cells in mouse skeletal muscle. PLoS One 7: e44791.
4. Imoukhuede PI, Popel AS (2011) Quantification and cell-to-cell variation of vascular endothelial growth factor receptors. Exp Cell Res 317: 955-965.
5. Mac Gabhann F, Popel AS (2007) Dimerization of VEGF receptors and implications for signal transduction: a computational study. Biophys Chem 128: 125-139.
6. Mac Gabhann F, Popel AS (2007) Interactions of VEGF isoforms with VEGFR-1, VEGFR-2, and neuropilin in vivo: a computational model of human skeletal muscle. Am J Physiol Heart Circ Physiol 292: H459-474.
7. Zhang XY, Birtwistle MR, Gallo JM (2014) A General Network Pharmacodynamic Model-Based Design Pipeline for Customized Cancer Therapy Applied to the VEGFR Pathway. CPT Pharmacometrics Syst Pharmacol 3: e92.
8. Carter TD, Ogden D (1997) Kinetics of Ca2+ release by InsP3 in pig single aortic endothelial cells: evidence for an inhibitory role of cytosolic Ca2+ in regulating hormonally evoked Ca2+ spikes. J Physiol 504 (Pt 1): 17-33.
9. Wiesner TF, Berk BC, Nerem RM (1996) A mathematical model of cytosolic calcium dynamics in human umbilical vein endothelial cells. Am J Physiol 270: C1556-1569.
10. De Young GW, Keizer J (1992) A single-pool inositol 1,4,5-trisphosphate-receptor-based model for agonist-stimulated oscillations in Ca2+ concentration. Proc Natl Acad Sci U S A 89: 9895-9899.
11. Pepke S, Kinzer-Ursem T, Mihalas S, Kennedy MB (2010) A dynamic model of interactions of Ca2+, calmodulin, and catalytic subunits of Ca2+/calmodulin-dependent protein kinase II. PLoS Comput Biol 6: e1000675.
12. Winslow RL, Scollan DF, Holmes A, Yung CK, Zhang J, et al. (2000) Electrophysiological modeling of cardiac ventricular function: from cell to organ. Annu Rev Biomed Eng 2: 119-155.
13. Bhalla US, Ram PT, Iyengar R (2002) MAP kinase phosphatase as a locus of flexibility in a mitogen-activated protein kinase signaling network. Science 297: 1018-1023.
14. Yamniuk AP, Nguyen LT, Hoang TT, Vogel HJ (2004) Metal ion binding properties and conformational states of calcium- and integrin-binding protein. Biochemistry 43: 2558-2568.
15. Luik RM, Wang B, Prakriya M, Wu MM, Lewis RS (2008) Oligomerization of STIM1 couples ER calcium depletion to CRAC channel activation. Nature 454: 538-542.
16. Schmeitz C, Hernandez-Vargas EA, Fliegert R, Guse AH, Meyer-Hermann M (2013) A mathematical model of T lymphocyte calcium dynamics derived from single transmembrane protein properties. Front Immunol 4: 277.
