# Supplementary material for: Computational investigation of sphingosine kinase 1 (SphK1) and calcium dependent ERK1/2 activation downstream of VEGFR2 in endothelial cells
Source: PLoS Comput Biol. 2017 Feb 8;13(2):e1005332. doi: 10.1371/journal.pcbi.1005332 (PMC5298229; doi:10.1371/journal.pcbi.1005332)
Supplement: S2 Table — (PDF) [file pcbi.1005332.s007.pdf]

Table S2. Initial values for the seed species in the model

Name	Value (units)
$PLC\gamma_0$	0.2 μM
$CaER_0$	2000 μM
$Calcium_0$	0.050 μM
CaF_0	118.03 μM
$CaFbound_0$	1.97 μM
CaM_0	1 μM
PKC_0	0.1 μM
$CIB1_0$	0.5 μM
$SphK1_0$	0.1 μM
Sph_0	10 μM
$S1P_0$	0 μM
$RasGDP_0$	0 μM
$RasGTP_0$	0 μM
Raf_0	0.355 μM
$MEK1/2_0$	0.289 μM
$ERK1/2_0$	0.382 μM
$ICRAC_0$ (initial CRAC channel current)	0.542 μM/s (0.094 pA)
