# Supplementary material for: Computational investigation of sphingosine kinase 1 (SphK1) and calcium dependent ERK1/2 activation downstream of VEGFR2 in endothelial cells
Source: PLoS Comput Biol. 2017 Feb 8;13(2):e1005332. doi: 10.1371/journal.pcbi.1005332 (PMC5298229; doi:10.1371/journal.pcbi.1005332)
Supplement: S1 Model Description — (PDF) [file pcbi.1005332.s008.pdf]

Seed species with description

1. **vegf(r,r,nrp1bd,c~s~i)**
   r: Binding sites for receptors
   nrp1bd: NRP1 binding domain
   c: Compartment, either surface (s) or internalized (i)
2. **vegfr2(l1,Y1175~Y~pY,dimer,c~s~i)**
   l1: Binding site for VEGF
   Y1175: Tyrosine 1175 either in unphosphorylated (Y) or phosphorylated state (pY)
   dimer: Ligand-independent coupling site
   c: Compartment, either surface(s) or internalized (i)
3. **vegfr1(l2,dimer,nrp1bd,c~s)**
   l2: Binding site for VEGF
   nrp1bd: NRP1 binding domain
   c: Compartment, only on the surface (s)
4. **NRP1(vegfabd,c~s~i)**
   vegfabd: VEGF binding domain
   c: Compartment, either surface (s) or internalized (i)
5. **PI(PIsite~3P~4P)**
   PIP2 on the membrane.
6. **PLCgamma(Yplc~Y~pY)**
   PLCγ in two state, either active (when Yplc is phosphorylated) or inactive (unphosphorylated Yplc)
7. **DAG(pkcbd)**
   DAG, with PKC binding domain (pkcbd)
8. **IP3_cyto(ip3rbd)**
   IP3 in cytoplasm with IP3R binding domain ip3rbd. The direct binding of IP3 to IP3R is not explicitly modelled in the current model, but is included to ease the extension of the model in the future.
9. **Calcium_cyto(bd)**
   Calcium in cytoplasm with a single binding domain (bd).
10. **CaER(bd)**
    Calcium within the ER lumen with a single binding domain (bd).
11. **CaF(cabd)**
    Free calcium buffer with single binding domain for calcium (cabd).
12. **CSQNF(cabd)**
    Free calcium buffer within the ER lumen with a single calcium binding domain (cabd)
13. **PKC(CalciumBD,DAGBD)**
    PKC with binding domain for calcium (CalciumBD) and DAG (DAGBD)
14. **CIB1(EF1,EF2,sk1bd,location~cytosol~membrane)**

CIB1 with two EF-hand domains (EF1 and EF2), with SphK1 binding domain (sk1bbd), with location either in cytosol or membrane.

15. **SphK(CIB1bd,Serk~S~pS)**
    SphK1 with CIB1 binding domain (CIB1bd) and S225 phosphorylation site (denoted by Serk)
16. **Sph(skbd)**
    Sphingosin with SphK1 binding domain (skbd).
17. **S1P(bd)**
    Sphingosin-1-phosphpate with a single binding domain (bd).
18. **RasGDP(rafbd)**
    RasGDP with a single binding domain (rafbd)
19. **RasGTP(rafbd)**
    RasGTP with a single binding domain (rafbd)
20. **Raf(mekbd,rasbd,Y1Y2~Y~pY,Spkc~S~pS)**
    Raf with a MEK1/2 binding domain (mekbd), RasGTP binding domain (rasbd), and tyrosine phosphorylation site (Y1Y2) and Serine phosphorylation site (Spkc)
21. **MEK12(bd,S1~S~pS,S2~S~pS)**
    MEE1/2 with a binding domain (bd), MEK1 Serine site (S1), MEK2 Serine site (S2).
22. **ERK1(MEK12bd,S1~S~pS)**
    ERK1 with MEK1/2 binding domain (MEK12bd) and a Serine site (S1)
23. **ERK2(MEK12bd,S2~S~pS)**
    ERK2 with MEK1/2 binding domain (MEK12bd) and a Serine site (S2)
24. **Istim()**
    Current through the CRAC channel.

Receptor phosphorylation rule

VEGFR2 homodimers containing VEGF undergo autophosphorylation with a rate kp_{Y1175}.

Receptors on the surface:

vegf(r!1,r!2,c~s).vegfr2(l1!1,c~s).vegfr2(l1!2,Y1175~Y,c~s) -> \

vegf(r!1,r!2,c~s).vegfr2(l1!1,c~s).vegfr2(l1!2,Y1175~pY,c~s) kpY1175

For internalized receptors:

vegf(r!1,r!2,c~i).vegfr2(l1!1,c~i).vegfr2(l1!2,Y1175~Y,c~i) -> \

vegf(r!1,r!2,c~i).vegfr2(l1!1,c~i).vegfr2(l1!2,Y1175~pY,c~i) kpY1175

Receptor dephosphorylation

Surface with rate kdps:

vegfr2(Y1175~**pY**,c~s) -> vegfr2(Y1175~**Y**,c~s) kdps

Endosomes with rate kdpi:

vegfr2(Y1175~**pY**,c~i) -> vegfr2(Y1175~**Y**,c~i) kdpi

Receptor internalization

Receptor complexes not containing NRP1 (nrp1bd free below) but with no coupling (the ligand-independent dimerization site free below)

vegf(r!1,r!2,nrp1bd,c~s).vegfr2(l1!1,dimer,c~s).vegfr2(l1!2,dimer,c~s) -> \

vegf(r!1,r!2,nrp1bd,c~i).vegfr2(l1!1,dimer,c~i).vegfr2(l1!2,dimer,c~i) kr2si

Receptor complexes not containing NRP1 (nrp1bd free below) but with no coupling (the ligand-independent dimerization site engaged below)

vegf(r!1,r!2,nrp1bd,c~s).vegfr2(l1!1,dimer!6,c~s).vegfr2(l1!2,dimer!6,c~s) -> \

vegf(r!1,r!2,nrp1bd,c~i).vegfr2(l1!1,dimer!6,c~i).vegfr2(l1!2,dimer!6,c~i) kr2si

Receptor complexes containing NRP1 (nrp1bd engaged below) but with no coupling (the ligand-independent dimerization site free below)

vegf(r!1,r!2,nrp1bd!9,c~s).NRP1(vegfabd!9,c~s).vegfr2(l1!1,dimer,c~s).vegfr2(l1!2,dimer,c~s) -> \

vegf(r!1,r!2,nrp1bd!9,c~i).NRP1(vegfabd!9,c~i).vegfr2(l1!1,dimer,c~i).vegfr2(l1!2,dimer,c~i) kr2NRP1si

Receptor complexes containing NRP1 (nrp1bd engaged below) but with coupling (the ligand-independent dimerization site engaged below)

vegf(r!1,r!2,nrp1bd!9,c~s).NRP1(vegfabd!9,c~s).vegfr2(l1!1,dimer!6,c~s).vegfr2(l1!2,dimer!6,c~s) -> \

vegf(r!1,r!2,nrp1bd!9,c~i).NRP1(vegfabd!9,c~i).vegfr2(l1!1,dimer!6,c~i).vegfr2(l1!2,dimer!6,c~i) kr2NRP1si

Single receptor internalization

Single VEGFR2 receptor not bound to VEGF with coupling domain free (dimer)

vegfr2(l1,dimer,c~s) -> vegfr2(l1,dimer,c~i) ksingleR2si

Single VEGFR2 receptor not bound to VEGF with coupling domain engaged (dimer)

vegfr2(l1,dimer!1,c~s).vegfr2(l1,dimer!1,c~s) -> vegfr2(l1,dimer!1,c~i).vegfr2(l1,dimer!1,c~i) ksingleR2si

Recycling of receptors

Note: Recycling of phosphorylated receptors turns out to be negligible in the model.

Receptor complexes not containing NRP1 (nrp1bd free below) but with no coupling (the ligand-independent dimerization site free below)

vegf(r!1,r!2,nrp1bd,c~i).vegfr2(l1!1,dimer,c~i).vegfr2(l1!2,dimer,c~i) -> \

vegf(r!1,r!2,nrp1bd,c~s).vegfr2(l1!1,dimer,c~s).vegfr2(l1!2,dimer,c~s) kr2is

Receptor complexes not containing NRP1 (nrp1bd free below) but with no coupling (the ligand-independent dimerization site engaged below)

vegf(r!1,r!2,nrp1bd,c~i).vegfr2(l1!1,dimer!6,c~i).vegfr2(l1!2,dimer!6,c~i) -> \

vegf(r!1,r!2,nrp1bd,c~s).vegfr2(l1!1,dimer!6,c~s).vegfr2(l1!2,dimer!6,c~s) kr2is

Receptor complexes containing NRP1 (nrp1bd engaged below) but with no coupling (the ligand-independent dimerization site free below)

vegf(r!1,r!2,nrp1bd!9,c~i).NRP1(vegfabd!9,c~i).vegfr2(l1!1,dimer,c~i).vegfr2(l1!2,dimer,c~i) -> \

vegf(r!1,r!2,nrp1bd!9,c~s).NRP1(vegfabd!9,c~s).vegfr2(l1!1,dimer,c~s).vegfr2(l1!2,dimer,c~s) kr2NRP1is

Receptor complexes containing NRP1 (nrp1bd engaged below) but with coupling (the ligand-independent dimerization site engaged below)

vegf(r!1,r!2,nrp1bd!9,c~i).NRP1(vegfabd!9,c~i).vegfr2(l1!1,dimer!6,c~i).vegfr2(l1!2,dimer!6,c~i) -> \vegf(r!1,r!2,nrp1bd!9,c~s).NRP1(vegfabd!9,c~s).vegfr2(l1!1,dimer!6,c~s).vegfr2(l1!2,dimer!6,c~s) kr2NRP1is

Single receptor cycling

Single VEGFR2 free of ligand with coupling site free (dimer)

vegfr2(l1,dimer,c~i) -> vegfr2(l1,dimer,c~s) ksingleR2is

Single VEGFR2 free of ligand with coupling site engaged (dimer)

vegfr2(l1,dimer!1,c~i).vegfr2(l1,dimer!1,c~i) -> vegfr2(l1,dimer!1,c~s).vegfr2(l1,dimer!1,c~s) ksingleR2is

Receptor degradation

Degradation of phosphorylated VEGFR2 (at least one VEGFR2 is phosphorylated in the complex) with no NRP1 bound, with degradation rate kdegi0

vegf(r!1,nrp1bd,c~i).vegfr2(l1!1,Y1175~pY,c~i) -> Trash() kdegi0

Degradation of unphosphorylated VEGFR2 with no NRP1 bound, with degradation rate kdegi0noUB

vegf(r!1,r!2,nrp1bd,c~i).vegfr2(l1!1,Y1175~Y,c~i).vegfr2(l1!2,Y1175~Y,c~i) -> Trash() kdegi0noUB

Degradation of single VEGFR2 with the coupling site (dimer) free, with degradation rate kdegi0noUB

vegfr2(l1,dimer,c~i) -> Trash() kdegi0noUB

Degradation of single VEGFR2 with the coupling site (dimer) engaged, with degradation rate kdegi0noUB

vegfr2(l1,dimer!1,c~i).vegfr2(l1,dimer!1,c~i) -> Trash() kdegi0noUB

Degradation of phosphorylated VEGFR2 (at least one VEGFR2 phosphorylated within the complex) with NRP1 bound, with degradation rate kdegr2NRP1i0

vegf(r!1,nrp1bd!+,c~i).vegfr2(l1!1,Y1175~pY,c~i) -> Trash() kdegr2NRP1i0

Degradation of unphosphorylated VEGFR2 with NRP1 bound, with degradation rate kdegr2NRP1i0noUB

vegf(r!1,r!2,nrp1bd!+,c~i).vegfr2(l1!1,Y1175~Y,c~i).vegfr2(l1!2,Y1175~Y,c~i) -> Trash() kdegr2NRP1i0noUB

Calcium cycling module

IP3 generation

$$\mathrm{PLC}_{\gamma} + \mathrm{PIP2} \rightarrow \mathrm{PLC}_{\gamma} + \mathrm{IP3} + \mathrm{DAG} \quad \text{rate}=\frac{kcat_{\mathrm{PLC}\gamma/\mathrm{DAG}}[\mathrm{PIP2}]^{n_{DAG}}}{\mathrm{km}_{PIP2PLC\gamma}{}^{n_{DAG}}+[\mathrm{PIP2}]^{n_{DAG}}}$$

$$IP3 \rightarrow \phi \quad \text{rate}=k_{\deg,IP3}[\mathrm{IP3}] \qquad (1)$$

$$DAG \rightarrow \phi \quad \text{rate}=k_{deg,DAG}[\mathrm{DAG}]$$

IP3 induced calcium elevation (equations are taken from [1])

$$\frac{d[Ca]_{cyto}}{dt}=J_{CRAC}+J_{IP3R}\frac{Vol_{ER}}{Vol_{cyto}}+J_{Leak,ER}-J_{SERCA}-J_{PMCA}\underbrace{-k_{B,on}(T-[B])[Ca]_{cyto}+k_{B,off}[B]}_{\text{Calcium buffering term in cytoplasm}}$$

(2)

$$\frac{d[Ca]_{ER}}{dt}=(-J_{IP3R}+(J_{SERCA}-J_{Leak,ER})\frac{Vol_{cyto}}{Vol_{ER}})\underbrace{\frac{1}{1+\frac{CSQN_{total}}{(Kd_{CSQN}+[Ca]_{ER})^2}}}_{\text{Calcium buffering inside ER}} \qquad (3)$$

$$J_{IP3R}=\bar{I}_{IP3R}\frac{[IP3]^{3.8}}{[IP3]^{3.8}+K_{m,IP3R}^{3.8}}\frac{K_{i,Ca}^{3.8}}{K_{i,Ca}^{3.8}+[Ca]_{cyto}^{3.8}}([Ca]_{ER}-[Ca]_{cyto}) \qquad (4)$$

$$J_{SERCA}=\bar{I}_{SERCA}\left(\frac{[Ca]_{cyto}}{[Ca]_{cyto}+K_{m,PMCA}}\right)^2 \qquad (5)$$

$$J_{PMCA}=I_{PMCA}\frac{[Ca]_{cyto}^{1.4}}{[Ca]_{cyto}^{1.4}+K^{1.4}{}_{m,PMCA}} \qquad (6)$$

$$J_{Leak,ER}=Kleak_{ER}([Ca]_{ER}-[Ca]_{cyto})^2 \qquad (7)$$

The description of the current flux through CRAC channels (J_{CRAC}) is a simplified version of the mathematical model developed by Schmeitz *et al.* in T-cells [2]. The current through the CRAC channels is activated to the steady-state level with a time constant τ_{CRAC}. The steady-state current as a function of calcium concentration within the ER lumen follows a Hill function experimentally determined by Luik *et al*. [3].

$$\frac{dJ_{CRAC}}{dt}=\frac{I_{CRAC}-J_{CRAC}}{\tau_{CRAC}} \qquad (8)$$

$$I_{\mathrm{CRAC}} = \frac{\overline{I}_{\mathrm{CRAC}} K_{\mathrm{CRAC}}^{4.2}}{K_{\mathrm{CRAC}}^{4.2} + [Ca]_{ER}^{4.2}} \quad (9)$$

SphK1 activation module

SphK1 phosphorylation by ERK2

$pERK2$ + SphK1 $\rightarrow$ pERK2 + pSphK1

- $$\text{Rate}=\frac{kcat_{ERK}[pERK2][SphK1]}{[SphK1]+Km_{ERKSK1}} \quad (10)$$

Dephosphorylation of SphK1

pSphK1 $\rightarrow$ SphK1

$$\text{Rate}=\text{kdp}_{\text{SK1}}[pSphK1] \quad (11)$$

Binding of calcium-bound CIB1 to pSphK1

CIB1.Ca + pSphK1$_{cyto}$ $\leftrightarrow$ CIB1.Ca.pSphK1$_{cyto}$ kon$_{\text{CIB1/SK1}}$,koff$_{\text{CIB1SK1}}$ (12)

SphK1 translocation from cytoplasm to the membrane

CIB1.Ca.pSphK1$_{cyto}$ $\leftrightarrow$ CIB1.Ca.pSphK1$_{membrane}$ kt$_{\text{SK1}}$, Ktoff$_{\text{SK1}}$ (13)

S1P generation at the membrane

CIB1.Ca.pSphK1$_{membrane}$ + Sph $\rightarrow$ S1P + CIB1.Ca.pSphK1$_{membrane}$

$$\text{Rate}=\frac{kcat_{SK1}[CIB1.Ca.pSphK1_{membrane}][Sph]}{[Sph]+Km_{SK1/Sph}} \quad (14)$$

S1P $\rightarrow$ Sph

$$\text{Rate} = kdp_{S1P}\ [S1P] \quad (15)$$

1. Silva HS, Kapela A, Tsoukias NM (2007) A mathematical model of plasma membrane electrophysiology and calcium dynamics in vascular endothelial cells. Am J Physiol Cell Physiol 293: C277-293.
2. Schmeitz C, Hernandez-Vargas EA, Fliegert R, Guse AH, Meyer-Hermann M (2013) A mathematical model of T lymphocyte calcium dynamics derived from single transmembrane protein properties. Front Immunol 4: 277.
3. Luik RM, Wang B, Prakriya M, Wu MM, Lewis RS (2008) Oligomerization of STIM1 couples ER calcium depletion to CRAC channel activation. Nature 454: 538-542.
