# Supplementary material for: Computational investigation of sphingosine kinase 1 (SphK1) and calcium dependent ERK1/2 activation downstream of VEGFR2 in endothelial cells
Source: PLoS Comput Biol. 2017 Feb 8;13(2):e1005332. doi: 10.1371/journal.pcbi.1005332 (PMC5298229; doi:10.1371/journal.pcbi.1005332)
Supplement: S1 BioNetGen Text — (DOCX) [file pcbi.1005332.s010.docx]

# Text of the BioNetGen file

begin model

begin parameters

Volcyto 9.12E-13

Volext 0.002

VolER 3.35E-13

fextmolar 1.205E+15

fcmolar 549000

cellarea 1400

VEGF165a_0 0.001190476

VEGFR2_0 4.29E+00

VEGFR1_0 1.428571429

NRP1_0 2.86E+01

Calcium_0 0.05010232

kvron 4.4

kvroff 2.60E-02

kcVR 0.002126712

kcRR 1.109768189

kdRR 0.784915318

kvronvegfr1 22

kvroffvegfr1 0.026

kdeltaRR 2.503952539

kdeltaVR 2.052324738

kpY1175 4239

kdps 769.0416257

kdpi 5.438985303

kr2si 0.060670655

kr2is 0.001242368

kr2NRP1si 0.404165983

kr2NRP1is 0.755627395

kVEGFNRP1on 3.2

kVEGFNRP1off 0.001

kNRP1VEGFR2on 0.553575486

kNRP1VEGFR2off 4.89126933

kNRP1VEGFR1on 1.314223544

kNRP1VEGFR1off 840.6509211

kVEGFR2NRP1on 0.005707355

kVEGFR2NRP1off 5.051782424

kVEGFR1NRP1on 0.015561472

kVEGFR1NRP1off 5.355945178

PIP2_0 10

kPIP2gen 0.000048

kSphgen 0.000048

kpPLCgamma 0.1

kdpPLCgamma 0.1

PLCgamma_0 0.2

kmPIP2PLCgamma 0.193585826

nDAG 2.495436003

kcatPLCgammaDAG 0.10046696

kdeg_ip3 0.0921875

kdeg_DAG 0.108898602

DAG_0 0

IP3_0 0

CaER_0 2.00E+03

Iip3Ramp 3.62E+04

KmIP3R 1.6

I_PMCAbar 5.978039695

KmPMCA 0.26

Caext 2000

KpmLeak 0

I_ERCA 4.76870897

KleakER 7.48E-08

KmERCA 0.15

KiCa 1

KaCa 0.1

KBon 100

KBoff 300

CaF_0 118.0327869

CaFbound_0 1.967213115

CSQN_total 15000

KCSQN 800

PKC_0 0.1

konCaPKC 0.3

koffCaPKC 0.01

konDAGPKC 0.029957319

koffDAGPKC 0.124096212

kon1CaCIB1 0.052631579

koff1CaCIB1 0.1

kon2CaCIB1 0.185185185

koff2CaCIB1 0.1

CIB1_0 0.5

konCIB1SphK1 17.6028519

koffCIB1SphK1 4.403956392

SphK_0 0.1

Sph_0 10

S1P_0 0

KmSK1Sph 0.029430478

kcatSK1Sph 37.23820691

ktSK1 1

koffSK1 1.04E-01

kdpSK1 0.02182773

RasGTP_0 0

RasGDP_0 0.1

kRasGAP 2.941097467

kdegi0 1.41E-03

Raf_0 0.355471965

MEK12_0 0.288919159

ERK12_0 0.382329627

konRasRaf 13.10183719

koffRasRaf 0.151878139

kpRaf 1.67558904

kdpRaf 0.894826206

kdpPKCRaf 0.720296112

KmMEK12Raf 0.807388937

kpMEK12Raf1 1.801786102

kdpMEK12_1 0.111827134

kdpMEK12_2 0.139705453

kmMEKERK12 0.25546079

kpMEK12ERK12_1 12.14930177

kdpERK12_1 6.06E+00

kdpERK12_2 1.053392443

kcatERK 7.882741692

kmERKSK1 1.198236617

kS1PRas 1.556423632

KmS1PRas 5.899306591

kdpS1P 1.188017664

kcatPKC 10.20798409

kmPKCRaf 0.313875333

ksingleR2syn 4.05E-08

ksingleR2si 9.23E-04

ICrac 1.74E+04

Kcrac 169

kpMEK12Raf2 1.204676328

kpMEK12ERK12_2 0.516270813

kdegr2NRP1i0 1.18E-02

tau_stim 4

kmPLCgammaR2 8

Istim0 0.542138034

ncrac 4.2

ktoffSK1 6.97E-04

kdegi0noUB 9.37E-04

kdegr2NRP1i0noUB 1.00E-02

ksingleR2is 0.267258037

end parameters

begin molecule types

vegf(r,r,nrp1bd,c~s~i) # vegf165a

vegfr2(l1,Y1175~Y~pY,dimer,c~s~i)

vegfr1(l2,dimer,nrp1bd,c~s)

NRP1(vegfabd,c~s~i)

PI(PIsite~3P~4P)

PLCgamma(Yplc~Y~pY)

DAG(pkcbd)

IP3_cyto(ip3rbd)

Calcium_cyto(bd)

Trash()

CaER(bd)

CaF(cabd)

CSQNF(cabd)

PKC(CalciumBD,DAGBD)

I()

CIB1(EF1,EF2,sk1bd,location~cytosol~membrane)

SphK(CIB1bd,Serk~S~pS)

Sph(skbd)

S1P(bd)

RasGDP(rafbd)

RasGTP(rafbd)

Raf(mekbd,rasbd,Y1Y2~Y~pY,Spkc~S~pS)

MEK12(bd,S1~S~pS,S2~S~pS)

ERK1(MEK12bd,S1~S~pS)

ERK2(MEK12bd,S2~S~pS)

Istim()

end molecule types

begin seed species

vegf(r,r,nrp1bd,c~s) VEGF165a_0

vegfr1(l2,dimer,nrp1bd,c~s) VEGFR1_0

vegfr2(l1,Y1175~Y,dimer,c~s) VEGFR2_0

NRP1(vegfabd,c~s) NRP1_0

PI(PIsite~3P) PIP2_0

PLCgamma(Yplc~Y) PLCgamma_0

CaER(bd) CaER_0

Calcium_cyto(bd) Calcium_0

CaF(cabd) CaF_0

Calcium_cyto(bd!1).CaF(cabd!1) CaFbound_0

PKC(CalciumBD,DAGBD) PKC_0

I() 1

CIB1(EF1,EF2,sk1bd,location~cytosol) CIB1_0

SphK(CIB1bd,Serk~S) SphK_0

Sph(skbd) Sph_0

S1P(bd) S1P_0

RasGDP(rafbd) RasGDP_0

RasGTP(rafbd) RasGTP_0

Raf(mekbd,rasbd,Y1Y2~Y,Spkc~S) Raf_0

MEK12(bd,S1~S,S2~S) MEK12_0

ERK1(MEK12bd,S1~S) ERK12_0

ERK2(MEK12bd,S2~S) ERK12_0

Istim() Istim0

end seed species

begin observables

Molecules VEGFR2tots vegfr2(c~s)

Molecules VEGFR2toti vegfr2(c~i)

Molecules VEGFR2total vegfr2()

Molecules vr2s vegfr2(l1,c~s)

Molecules vr2i vegfr2(l1,c~i)

Molecules vr1s vegfr1(l2,nrp1bd,c~s)

Molecules vegffrees vegf(r,r,nrp1bd,c~s)

Molecules vegfr2Y1175ps vegfr2(Y1175~pY!?,c~s)

Molecules vegfr2Y1175pi vegfr2(Y1175~pY!?,c~i)

Molecules r2Y1175total vegfr2(Y1175~pY!?)

Molecules vegfr1tots vegfr1(c~s)

Molecules NRP1VEGFR2s vegf(r!1,r!2,nrp1bd!+,c~s).vegfr2(l1!1,c~s).vegfr2(l1!2,c~s)

Molecules NRP1VEGFR2i vegf(r!1,r!2,nrp1bd!+,c~i).vegfr2(l1!1,c~i).vegfr2(l1!2,c~i)

Molecules freeDAGs DAG(pkcbd!?)

Molecules activePLCgamma PLCgamma(Yplc~pY!?)

Molecules PIP2 PI(PIsite~3P)

Molecules PIP3 PI(PIsite~4P)

Molecules freeip3cyto IP3_cyto(ip3rbd)

Molecules Cac Calcium_cyto(bd)

Molecules Caer CaER(bd)

Molecules CaBuf_fer CaF(cabd!+)

Molecules activePKCs PKC(CalciumBD!+,DAGBD!1).DAG(pkcbd!1)

Molecules activePKCtot PKC(CalciumBD!+,DAGBD!+)

Molecules SphKpkc SphK(Serk~pS!?)

Molecules S1phosphate S1P(bd)

Molecules NRP1frees NRP1(vegfabd,c~s)

Molecules NRP1freei NRP1(vegfabd,c~i)

Molecules NRP1bounds NRP1(vegfabd!+,c~s)

Molecules NRP1boundi NRP1(vegfabd!+,c~i)

Molecules vr2dimers vegfr2(l1,dimer!1,c~s).vegfr2(l1,dimer!1,c~s)

Molecules vr2dimeri vegfr2(l1,dimer!1,c~i).vegfr2(l1,dimer!1,c~i)

Molecules NRP1totals NRP1(c~s)

Molecules NRP1totali NRP1(c~i)

Molecules singleNRP1totals NRP1(vegfabd,c~s)

Molecules singleNRP1totali NRP1(vegfabd,c~i)

Molecules vegfr2total vegf(r!1).vegfr2(l1!1)

Molecules vr2Y1175s vegfr2(Y1175~pY,c~s)

Molecules vr2Y1175i vegfr2(Y1175~pY,c~i)

Molecules plcgammafree PLCgamma(Yplc~Y)

Molecules rasgdpfree RasGDP(rafbd!?)

Molecules rasgtpfree RasGTP(rafbd!?)

Molecules activeRafbyrastot Raf(Y1Y2~pY!?)

Molecules phosphoMEK12tot MEK12(S1~pS!?,S2~pS!?)

Molecules phosphoERK1tot ERK1(S1~pS!?)

Molecules phosphoERK2tot ERK2(S2~pS!?)

Molecules SphK1 SphK(CIB1bd!+,Serk~S).CIB1(sk1bd!1,location~membrane)

Molecules SphK1mempS SphK(CIB1bd!+,Serk~pS).CIB1(sk1bd!1,location~membrane)

Molecules SphK1cytosol SphK(CIB1bd!+,Serk~S).CIB1(sk1bd!1,location~cytosol)

Molecules activeSphK1 SphK(Serk~pS!?)

Molecules freeSK1 SphK(CIB1bd,Serk~S)

Molecules freeSK1mem SphK(CIB1bd!1,Serk~S).CIB1(sk1bd!1,location~membrane)

Molecules freecib1 CIB1(EF1,EF2,sk1bd)

Molecules calciumcib1 CIB1(EF1!+,EF2!+,sk1bd)

Molecules sk1bcib1 CIB1(EF1!+,EF2!+,sk1bd!+)

Molecules nrp1s NRP1(vegfabd,c~s)

Molecules vegfnrp1s vegf(r,r,nrp1bd!1,c~s).NRP1(vegfabd!1,c~s)

Molecules vegfr1s vegf(r!1,r,nrp1bd,c~s).vegfr1(l2!1,c~s)

Molecules vegfr2s vegf(r!1,r,nrp1bd,c~s).vegfr2(l1!1,dimer,c~s)

Molecules freepip2 PI(PIsite~3P)

Molecules freesphingosin Sph(skbd)

Molecules freeraf Raf(Spkc~S)

Molecules activeRafPKC Raf(Spkc~pS!?)

Molecules activeRafPKCERK1 Raf(Spkc~pS!?,Y1Y2~pY!?)

Molecules activeRafPKCERK2 Raf(Spkc~pS!?,Y1Y2~Y!?)

Molecules activeRafPKCERK3 Raf(Spkc~S!?,Y1Y2~pY!?)

Molecules rafY1Y2pY Raf(Y1Y2~pY,Spkc~S)

Molecules rafY1Y2pYpS Raf(Y1Y2~pY,Spkc~pS)

Molecules rafpS Raf(Y1Y2~Y,Spkc~pS)

Molecules erk1s ERK1(S1~S)

Molecules mek12s MEK12(S1~S,S2~S)

Molecules R2singlei vegfr2(l1,Y1175~Y,dimer,c~i)

Molecules pERK1s ERK1(S1~pS)

Molecules mek12ps MEK12(S1~pS,S2~S)

Molecules pERK2s ERK2(S2~pS)

Molecules mek12ps1 MEK12(S1~pS)

Molecules mek12ps2 MEK12(S2~pS)

Molecules erk1ps ERK1(S1~pS)

Molecules erk2ps ERK2(S2~pS)

Molecules vr2r2py1175s vegfr2(Y1175~pY,c~s)

Molecules vr2r2py1175i vegfr2(Y1175~pY,c~i)

Species vr2py1175s vegf(r!1,r,c~s).vegfr2(l1!1,Y1175~pY,c~s)

Species vr2py1175i vegf(r!1,r,c~i).vegfr2(l1!1,Y1175~pY,c~i)

Molecules r2singlepy1175s vegfr2(l1,Y1175~pY,c~s)

Molecules r2singlepy1175i vegfr2(l1,Y1175~pY,c~i)

Molecules vr1r2pY1165s vegf(r!1,r!2,c~s).vegfr1(l2!1,c~s).vegfr2(l1!2,Y1175~pY,c~s)

Molecules pSK1 SphK(Serk~pS)

Molecules frees1p S1P(bd)

Molecules py1y2rafs Raf(Y1Y2~pY)

Molecules vegfnrp1i vegf(r,r,nrp1bd!1,c~i).NRP1(vegfabd!1,c~i)

Species vegfr2i vegf(r!1,r,nrp1bd,c~i).vegfr2(l1!1,c~i)

Molecules totalnrp1 NRP1(vegfabd)

Molecules pplcgamma PLCgamma(Yplc~pY)

Molecules phosphoERKpS1 ERK1(S1~pS!?)

Molecules phosphoERKpS2 ERK2(S2~pS!?)

Molecules phosphoMEKpS1 MEK12(S1~pS!?,S2~S)

Molecules phosphoMEKpS2 MEK12(S1~S,S2~pS!?)

Molecules freeSphK1 SphK(CIB1bd,Serk~S)

Molecules rafpkc Raf(Spkc~pS)

Molecules mek12s1 MEK12(S1~S)

Molecules mek12s2 MEK12(S2~S)

Molecules erk12s1 ERK1(S1~S)

Molecules erk12s2 ERK2(S2~S)

Molecules yplcgamma PLCgamma(Yplc~Y)

Molecules gtpfreeras RasGTP(rafbd)

Molecules bvegfr2 vegf(r!1,nrp1bd,c~s).vegfr2(l1!1,c~s)

Molecules bnrp1 vegf(nrp1bd!1,c~s).NRP1(vegfabd!1,c~s)

Molecules bvegfr1 vegf(r!1,nrp1bd,c~s).vegfr1(l2!1,c~s)

Molecules bvegfr1dimer vegf(r!1,r!2,c~s).vegfr1(l2!1,c~s).vegfr1(l2!2,c~s)

Molecules bvegfr2_2 vegf(r!1,c~s).vegfr2(l1!1,c~s)

Molecules bvegfr1_2 vegf(r!1,c~s).vegfr1(l2!1,c~s)

Molecules vegfbound_1 vegf(r!+,nrp1bd,c~s)

Species vegfbound_2 vegf(r,nrp1bd!+,c~s)

Molecules vegfbound_3 vegf(r!+,c~s)

Species vegfbound_4 vegf(r,r,nrp1bd!1,c~s).NRP1(vegfabd!1,c~s)

Molecules Iopenstim Istim()

Molecules nrp1r1s NRP1(vegfabd!1,c~s).vegfr1(nrp1bd!1,c~s)

Molecules CIB1mem CIB1(location~membrane)

end observables

begin reaction rules

# Ligand-independent coupling of the receptors

vegfr2(l1,dimer,c~s) + vegfr2(l1,dimer,c~s) <-> \

vegfr2(l1,dimer!1,c~s).vegfr2(l1,dimer!1,c~s) kcRR,kdRR

vegfr2(l1,dimer,c~s) + vegfr1(l2,dimer,c~s) <-> \

vegfr2(l1,dimer!1,c~s).vegfr1(l2,dimer!1,c~s) kcRR,kdRR

vegfr1(l2,dimer,c~s) + vegfr1(l2,dimer,c~s) <-> \

vegfr1(l2,dimer!1,c~s).vegfr1(l2,dimer!1,c~s) kcRR,kdRR

vegf(r!1,r!2,c~s).vegfr2(l1!1,dimer,c~s).vegfr2(l1!2,dimer,c~s) <-> \

vegf(r!1,r!2,c~s).vegfr2(l1!1,dimer!3,c~s).vegfr2(l1!2,dimer!3,c~s) kdeltaRR,kdRR

vegf(r!1,r!2,c~s).vegfr2(l1!1,dimer,c~s).vegfr1(l2!2,dimer,c~s) <-> \

vegf(r!1,r!2,c~s).vegfr2(l1!1,dimer!3,c~s).vegfr1(l2!2,dimer!3,c~s) kdeltaRR,kdRR

vegf(r!1,r!2,c~s).vegfr1(l2!1,dimer,c~s).vegfr1(l2!2,dimer,c~s) <-> \

vegf(r!1,r!2,c~s).vegfr1(l2!1,dimer!3,c~s).vegfr1(l2!2,dimer!3,c~s) kdeltaRR,kdRR

# VEGFR1 binding to NRP1

vegfr1(nrp1bd,c~s) + NRP1(vegfabd,c~s) <-> vegfr1(nrp1bd!1,c~s).NRP1(vegfabd!1,c~s) kNRP1VEGFR1on,kNRP1VEGFR1off

#Binding of VEGF165a to NRP1

I() -> I() + vegf(r,r,nrp1bd,c~s) -(kVEGFNRP1on*cellarea/fextmolar)*vegffrees*nrp1s+(kVEGFNRP1off*cellarea/fextmolar)*vegfnrp1s

vegf(r,r,nrp1bd,c~s) + NRP1(vegfabd,c~s) -> vegf(r,r,nrp1bd,c~s) + vegf(r,r,nrp1bd!1,c~s).NRP1(vegfabd!1,c~s) kVEGFNRP1on

vegf(r,r,nrp1bd!1,c~s).NRP1(vegfabd!1,c~s) -> NRP1(vegfabd,c~s) kVEGFNRP1off

vegf(r,r,nrp1bd!+,c~s) + vegfr2(l1,dimer,c~s) <-> \

vegf(r!2,r,nrp1bd!+,c~s).vegfr2(l1!2,dimer,c~s) kNRP1VEGFR2on,kNRP1VEGFR2off

vegf(r,r,nrp1bd!+,c~s) + vegfr2(l1,dimer!3,c~s).vegfr2(l1,dimer!3,c~s) <-> \

vegf(r!2,r,nrp1bd!+,c~s).vegfr2(l1!2,dimer!3,c~s).vegfr2(l1,dimer!3,c~s) kNRP1VEGFR2on,kNRP1VEGFR2off

vegf(r,r,nrp1bd!+,c~s) + vegfr2(l1,dimer!3,c~s).vegfr1(l2,dimer!3,c~s) <-> \

vegf(r!2,r,nrp1bd!+,c~s).vegfr2(l1!2,dimer!3,c~s).vegfr1(l2,dimer!3,c~s) kNRP1VEGFR2on,kNRP1VEGFR2off

vegf(r,r,nrp1bd!+,c~s) + vegfr1(l2,dimer,c~s) <-> \

vegf(r!2,r,nrp1bd!+,c~s).vegfr1(l2!2,dimer,c~s) kcVR,kvroffvegfr1

vegf(r,r,nrp1bd!+,c~s) + vegfr1(l2,dimer!3,c~s).vegfr1(l2,dimer!3,c~s) <-> \

vegf(r!2,r,nrp1bd!+,c~s).vegfr1(l2!2,dimer!3,c~s).vegfr1(l2,dimer!3,c~s) kcVR,kvroffvegfr1

vegf(r,r,nrp1bd!+,c~s) + vegfr1(l2,dimer!3,c~s).vegfr2(l1,dimer!3,c~s) <-> \

vegf(r!2,r,nrp1bd!+,c~s).vegfr1(l2!2,dimer!3,c~s).vegfr2(l1,dimer!3,c~s) kcVR,kvroffvegfr1

# Binding of VEGF165a to VEGFR2 receptors

I() -> I() + vegf(r,r,nrp1bd,c~s) -(kvron*cellarea/fextmolar)*vegffrees*vr2s+(kvroff*cellarea/fextmolar)*vegfr2s

vegf(r,r,nrp1bd,c~s) + vegfr2(l1,c~s) -> \

vegf(r,r,nrp1bd,c~s) + vegf(r!1,r,nrp1bd,c~s).vegfr2(l1!1,c~s) kvron

vegf(r!1,r,nrp1bd,c~s).vegfr2(l1!1,c~s) -> vegfr2(l1,c~s) kvroff

vegf(r!1,r,nrp1bd,c~s).vegfr2(l1!1,dimer,c~s) + vegfr2(l1,dimer,c~s) <-> \

vegf(r!1,r!2,nrp1bd,c~s).vegfr2(l1!1,dimer,c~s).vegfr2(l1!2,dimer,c~s) kcVR,kvroff

vegf(r!1,r,nrp1bd,c~s).vegfr2(l1!1,dimer,c~s) + vegfr1(l2,dimer,c~s) <-> \

vegf(r!1,r!2,nrp1bd,c~s).vegfr2(l1!1,dimer,c~s).vegfr1(l2!2,dimer,c~s) kcVR,kvroffvegfr1

vegf(r!1,r,nrp1bd,c~s).vegfr2(l1!1,dimer!3,c~s).vegfr2(l1,dimer!3,c~s) <-> \

vegf(r!1,r!2,nrp1bd,c~s).vegfr2(l1!1,dimer!3,c~s).vegfr2(l1!2,dimer!3,c~s) kdeltaVR,kvroff

vegf(r!1,r,nrp1bd,c~s).vegfr2(l1!1,dimer!3,c~s).vegfr1(l2,dimer!3,c~s) <-> \

vegf(r!1,r!2,nrp1bd,c~s).vegfr2(l1!1,dimer!3,c~s).vegfr1(l2!2,dimer!3,c~s) kdeltaVR,kvroffvegfr1

vegf(r!1,r,nrp1bd,c~s).vegfr1(l2!1,dimer,c~s) + vegfr1(l2,dimer,c~s) <-> \

vegf(r!1,r!2,nrp1bd,c~s).vegfr1(l2!1,dimer,c~s).vegfr1(l2!2,dimer,c~s) kcVR,kvroffvegfr1

vegf(r!1,r,nrp1bd,c~s).vegfr1(l2!1,dimer,c~s) + vegfr2(l1,dimer,c~s) <-> \

vegf(r!1,r!2,nrp1bd,c~s).vegfr1(l2!1,dimer,c~s).vegfr2(l1!2,dimer,c~s) kcVR,kvroff

vegf(r!1,r,nrp1bd,c~s).vegfr1(l2!1,dimer!3,c~s).vegfr1(l2,dimer!3,c~s) <-> \

vegf(r!1,r!2,nrp1bd,c~s).vegfr1(l2!1,dimer!3,c~s).vegfr1(l2!2,dimer!3,c~s) kdeltaVR,kvroffvegfr1

vegf(r!1,r,nrp1bd,c~s).vegfr1(l2!1,dimer!3,c~s).vegfr2(l1,dimer!3,c~s) <-> \

vegf(r!1,r!2,nrp1bd,c~s).vegfr1(l2!1,dimer!3,c~s).vegfr2(l1!2,dimer!3,c~s) kdeltaVR,kvroff

vegf(r!1,r,nrp1bd!+,c~s).vegfr2(l1!1,dimer,c~s) + vegfr2(l1,dimer,c~s) <-> \

vegf(r!1,r!2,nrp1bd!+,c~s).vegfr2(l1!1,dimer,c~s).vegfr2(l1!2,dimer,c~s) kNRP1VEGFR2on,kNRP1VEGFR2off

vegf(r!1,r,nrp1bd!+,c~s).vegfr2(l1!1,dimer,c~s) + vegfr1(l2,dimer,c~s) <-> \

vegf(r!1,r!2,nrp1bd!+,c~s).vegfr2(l1!1,dimer,c~s).vegfr1(l2!2,dimer,c~s) kcVR,kvroffvegfr1

vegf(r!1,r,nrp1bd!+,c~s).vegfr2(l1!1,dimer!3,c~s).vegfr2(l1,dimer!3,c~s) <-> \

vegf(r!1,r!2,nrp1bd!+,c~s).vegfr2(l1!1,dimer!3,c~s).vegfr2(l1!2,dimer!3,c~s) kdeltaVR,kvroff

vegf(r!1,r,nrp1bd!+,c~s).vegfr2(l1!1,dimer!3,c~s).vegfr1(l2,dimer!3,c~s) <-> \

vegf(r!1,r!2,nrp1bd!+,c~s).vegfr2(l1!1,dimer!3,c~s).vegfr1(l2!2,dimer!3,c~s) kdeltaVR,kvroffvegfr1

vegf(r!1,r,nrp1bd!+,c~s).vegfr1(l2!1,dimer,c~s) + vegfr1(l2,dimer,c~s) <-> \

vegf(r!1,r!2,nrp1bd!+,c~s).vegfr1(l2!1,dimer,c~s).vegfr1(l2!2,dimer,c~s) kcVR,kvroffvegfr1

vegf(r!1,r,nrp1bd!+,c~s).vegfr1(l2!1,dimer,c~s) + vegfr2(l1,dimer,c~s) <-> \

vegf(r!1,r!2,nrp1bd!+,c~s).vegfr1(l2!1,dimer,c~s).vegfr2(l1!2,dimer,c~s) kNRP1VEGFR2on,kNRP1VEGFR2off

vegf(r!1,r,nrp1bd!+,c~s).vegfr1(l2!1,dimer!3,c~s).vegfr1(l2,dimer!3,c~s) <-> \

vegf(r!1,r!2,nrp1bd!+,c~s).vegfr1(l2!1,dimer!3,c~s).vegfr1(l2!2,dimer!3,c~s) kdeltaVR,kvroffvegfr1

vegf(r!1,r,nrp1bd!+,c~s).vegfr1(l2!1,dimer!3,c~s).vegfr2(l1,dimer!3,c~s) <-> \

vegf(r!1,r!2,nrp1bd!+,c~s).vegfr1(l2!1,dimer!3,c~s).vegfr2(l1!2,dimer!3,c~s) kdeltaVR,kvroff

vegf(r!1,r!2,nrp1bd,c~s).vegfr2(l1!1,dimer!3,c~s).vegfr2(l1!2,dimer!3,c~s) + NRP1(vegfabd,c~s) <-> \

vegf(r!1,r!2,nrp1bd!4,c~s).vegfr2(l1!1,dimer!3,c~s).vegfr2(l1!2,dimer!3,c~s).NRP1(vegfabd!4,c~s) kVEGFR2NRP1on,kVEGFR2NRP1off

vegf(r!1,r!2,nrp1bd,c~s).vegfr1(l2!1,dimer!3,c~s).vegfr1(l2!2,dimer!3,c~s) + NRP1(vegfabd,c~s) <-> \

vegf(r!1,r!2,nrp1bd!4,c~s).vegfr1(l2!1,dimer!3,c~s).vegfr1(l2!2,dimer!3,c~s).NRP1(vegfabd!4,c~s) kVEGFR2NRP1on,kVEGFR2NRP1off

vegf(r!1,r!2,nrp1bd,c~s).vegfr2(l1!1,dimer!3,c~s).vegfr1(l2!2,dimer!3,c~s) + NRP1(vegfabd,c~s) <-> \

vegf(r!1,r!2,nrp1bd!4,c~s).vegfr2(l1!1,dimer!3,c~s).vegfr1(l2!2,dimer!3,c~s).NRP1(vegfabd!4,c~s) kVEGFR2NRP1on,kVEGFR2NRP1off

vegf(r!1,r!2,nrp1bd,c~s).vegfr2(l1!1,dimer,c~s).vegfr2(l1!2,dimer,c~s) + NRP1(vegfabd,c~s) <-> \

vegf(r!1,r!2,nrp1bd!4,c~s).vegfr2(l1!1,dimer,c~s).vegfr2(l1!2,dimer,c~s).NRP1(vegfabd!4,c~s) kVEGFR2NRP1on,kVEGFR2NRP1off

vegf(r!1,r,nrp1bd,c~s).vegfr2(l1!1,c~s) + NRP1(vegfabd,c~s) <-> vegf(r!1,r,nrp1bd!2,c~s).vegfr2(l1!1,c~s).NRP1(vegfabd!2,c~s) kVEGFR2NRP1on,kVEGFR2NRP1off

vegf(r!1,r,nrp1bd,c~s).vegfr1(l2!1,c~s) + NRP1(vegfabd,c~s) <-> vegf(r!1,r,nrp1bd!2,c~s).vegfr1(l2!1,c~s).NRP1(vegfabd!2,c~s) kVEGFR1NRP1on,kVEGFR1NRP1off

########################

# vegf165a binding to vegfr1

I() -> I() + vegf(r,r,nrp1bd,c~s) -kvronvegfr1*cellarea/fextmolar*vegffrees*vr1s+kvroffvegfr1*vegfr1s*(cellarea/fextmolar)

vegf(r,r,nrp1bd,c~s) + vegfr1(l2,c~s) -> \

vegf(r,r,nrp1bd,c~s) + vegf(r!1,r,nrp1bd,c~s).vegfr1(l2!1,c~s) kvron

vegf(r!1,r,nrp1bd,c~s).vegfr1(l2!1,c~s) -> vegfr1(l2,c~s) kvroffvegfr1

vegf(r!1,r,c~s).vegfr1(l2!1,dimer,c~s) + vegfr1(l2,dimer,c~s) <-> \

vegf(r!1,r!2,c~s).vegfr1(l2!1,dimer,c~s).vegfr1(l2!2,dimer,c~s) kcVR,kvroffvegfr1

vegf(r!1,r,c~s).vegfr1(l2!1,dimer,c~s) + vegfr2(l1,dimer,c~s) <-> \

vegf(r!1,r!2,c~s).vegfr1(l2!1,dimer,c~s).vegfr2(l1!2,dimer,c~s) kcVR,kvroff

vegf(r!1,r,c~s).vegfr1(l2!1,dimer!3,c~s).vegfr1(l2,dimer!3,c~s) <-> \

vegf(r!1,r!2,c~s).vegfr1(l2!1,dimer!3,c~s).vegfr1(l2!2,dimer!3,c~s) kdeltaVR,kvroffvegfr1

vegf(r!1,r,c~s).vegfr1(l2!1,dimer!3,c~s).vegfr2(l1,dimer!3,c~s) <-> \

vegf(r!1,r!2,c~s).vegfr1(l2!1,dimer!3,c~s).vegfr2(l1!2,dimer!3,c~s) kdeltaVR,kvroff

#################################

# Receptor phosphorylation

vegf(r!1,r!2,c~s).vegfr2(l1!1,c~s).vegfr2(l1!2,Y1175~Y,c~s) -> \

vegf(r!1,r!2,c~s).vegfr2(l1!1,c~s).vegfr2(l1!2,Y1175~pY,c~s) kpY1175

vegf(r!1,r!2,c~i).vegfr2(l1!1,c~i).vegfr2(l1!2,Y1175~Y,c~i) -> \

vegf(r!1,r!2,c~i).vegfr2(l1!1,c~i).vegfr2(l1!2,Y1175~pY,c~i) kpY1175

# Dephosphorylation of VEGFR2 species

vegfr2(Y1175~pY,c~s) -> vegfr2(Y1175~Y,c~s) kdps

vegfr2(Y1175~pY,c~i) -> vegfr2(Y1175~Y,c~i) kdpi

# Internalization s to i

vegf(r!1,r!2,nrp1bd,c~s).vegfr2(l1!1,dimer,c~s).vegfr2(l1!2,dimer,c~s) -> \

vegf(r!1,r!2,nrp1bd,c~i).vegfr2(l1!1,dimer,c~i).vegfr2(l1!2,dimer,c~i) kr2si

vegf(r!1,r!2,nrp1bd,c~s).vegfr2(l1!1,dimer!6,c~s).vegfr2(l1!2,dimer!6,c~s) -> \

vegf(r!1,r!2,nrp1bd,c~i).vegfr2(l1!1,dimer!6,c~i).vegfr2(l1!2,dimer!6,c~i) kr2si

vegf(r!1,r!2,nrp1bd!9,c~s).NRP1(vegfabd!9,c~s).vegfr2(l1!1,dimer,c~s).vegfr2(l1!2,dimer,c~s) -> \

vegf(r!1,r!2,nrp1bd!9,c~i).NRP1(vegfabd!9,c~i).vegfr2(l1!1,dimer,c~i).vegfr2(l1!2,dimer,c~i) kr2NRP1si

vegf(r!1,r!2,nrp1bd!9,c~s).NRP1(vegfabd!9,c~s).vegfr2(l1!1,dimer!6,c~s).vegfr2(l1!2,dimer!6,c~s) -> \

vegf(r!1,r!2,nrp1bd!9,c~i).NRP1(vegfabd!9,c~i).vegfr2(l1!1,dimer!6,c~i).vegfr2(l1!2,dimer!6,c~i) kr2NRP1si

# Recycling i to s

vegf(r!1,r!2,nrp1bd,c~i).vegfr2(l1!1,dimer,c~i).vegfr2(l1!2,dimer,c~i) -> \

vegf(r!1,r!2,nrp1bd,c~s).vegfr2(l1!1,dimer,c~s).vegfr2(l1!2,dimer,c~s) kr2is

vegf(r!1,r!2,nrp1bd,c~i).vegfr2(l1!1,dimer!6,c~i).vegfr2(l1!2,dimer!6,c~i) -> \

vegf(r!1,r!2,nrp1bd,c~s).vegfr2(l1!1,dimer!6,c~s).vegfr2(l1!2,dimer!6,c~s) kr2is

vegf(r!1,r!2,nrp1bd!9,c~i).NRP1(vegfabd!9,c~i).vegfr2(l1!1,dimer,c~i).vegfr2(l1!2,dimer,c~i) -> \

vegf(r!1,r!2,nrp1bd!9,c~s).NRP1(vegfabd!9,c~s).vegfr2(l1!1,dimer,c~s).vegfr2(l1!2,dimer,c~s) kr2NRP1is

vegf(r!1,r!2,nrp1bd!9,c~i).NRP1(vegfabd!9,c~i).vegfr2(l1!1,dimer!6,c~i).vegfr2(l1!2,dimer!6,c~i) -> \

vegf(r!1,r!2,nrp1bd!9,c~s).NRP1(vegfabd!9,c~s).vegfr2(l1!1,dimer!6,c~s).vegfr2(l1!2,dimer!6,c~s) kr2NRP1is

##################################################################################################

vegfr2(l1,dimer,c~i) -> vegfr2(l1,dimer,c~s) ksingleR2is

vegfr2(l1,dimer!1,c~i).vegfr2(l1,dimer!1,c~i) -> vegfr2(l1,dimer!1,c~s).vegfr2(l1,dimer!1,c~s) ksingleR2is

##################################################################################################

# Receptor degradation

vegf(r!1,nrp1bd,c~i).vegfr2(l1!1,Y1175~pY,c~i) -> Trash() kdegi0

vegf(r!1,r!2,nrp1bd,c~i).vegfr2(l1!1,Y1175~Y,c~i).vegfr2(l1!2,Y1175~Y,c~i) -> Trash() kdegi0noUB

vegfr2(l1,dimer,c~i) -> Trash() kdegi0noUB

vegfr2(l1,dimer!1,c~i).vegfr2(l1,dimer!1,c~i) -> Trash() kdegi0noUB

vegf(r!1,nrp1bd!+,c~i).vegfr2(l1!1,Y1175~pY,c~i) -> Trash() kdegr2NRP1i0

vegf(r!1,r!2,nrp1bd!+,c~i).vegfr2(l1!1,Y1175~Y,c~i).vegfr2(l1!2,Y1175~Y,c~i) -> Trash() kdegr2NRP1i0noUB

# single receptor cycling

vegfr2(l1,dimer,c~s) -> vegfr2(l1,dimer,c~i) ksingleR2si

vegfr2(l1,dimer!1,c~s).vegfr2(l1,dimer!1,c~s) -> vegfr2(l1,dimer!1,c~i).vegfr2(l1,dimer!1,c~i) ksingleR2si

# Activating PLCgamma

PLCgamma(Yplc~Y) + vegfr2(Y1175~pY,c~s) -> PLCgamma(Yplc~pY) + vegfr2(Y1175~pY,c~s) kpPLCgamma/(kmPLCgammaR2+yplcgamma)

PLCgamma(Yplc~Y) + vegfr2(Y1175~pY,c~i) -> PLCgamma(Yplc~pY) + vegfr2(Y1175~pY,c~i) kpPLCgamma/(kmPLCgammaR2+yplcgamma)

PLCgamma(Yplc~pY) -> PLCgamma(Yplc~Y) kdpPLCgamma

# IP3 and DAG generation

PLCgamma(Yplc~pY) + PI(PIsite~3P) -> IP3_cyto(ip3rbd) + PLCgamma(Yplc~pY) kcatPLCgammaDAG*freepip2^(nDAG-1)/(kmPIP2PLCgamma^nDAG+freepip2^nDAG)

PLCgamma(Yplc~pY) + PI(PIsite~3P) -> DAG(pkcbd) + PLCgamma(Yplc~pY) kcatPLCgammaDAG*freepip2^(nDAG-1)/(kmPIP2PLCgamma^nDAG+freepip2^nDAG)

I() -> I() + PI(PIsite~3P) kPIP2gen

IP3_cyto(ip3rbd) -> Trash() kdeg_ip3

DAG(pkcbd) -> Trash() kdeg_DAG

Calcium_cyto(bd) + CaF(cabd) <-> Calcium_cyto(bd!1).CaF(cabd!1) KBon,KBoff

I() -> I() + Istim() ICrac*(Kcrac^ncrac/(Kcrac^ncrac+Caer^ncrac))/tau_stim-Iopenstim/tau_stim

I() -> I() + Calcium_cyto(bd) (VolER/Volcyto)*Iip3Ramp*(Caer-Cac)*(freeip3cyto^3.8/(freeip3cyto^3.8+KmIP3R^3.8))*(KiCa^3.8/(KiCa^3.8+Cac^3.8))

I() -> I() + CaER(bd) -Iip3Ramp*(Caer-Cac)*(freeip3cyto^3.8/(freeip3cyto^3.8+KmIP3R^3.8))*(KiCa^3.8/(KiCa^3.8+Cac^3.8))*( 1/(1+CSQN_total/(KCSQN+Caer)^2) )

I() -> I() + Calcium_cyto(bd) -I_PMCAbar*Cac^1.4/(KmPMCA^1.4+Cac^1.4) + Iopenstim # PMCA pump

I() -> I() + Calcium_cyto(bd) -I_ERCA*(Cac/(KmERCA+Cac))^2 + KleakER*(Caer-Cac)^2 # SERCA pump

I() -> I() + CaER(bd) I_ERCA*(Cac/(KmERCA+Cac))^2*(Volcyto/VolER)*(1/(1+CSQN_total/(KCSQN+Caer)^2)) # SERCA pump

I() -> I() + CaER(bd) -KleakER*(Volcyto/VolER)*((Caer-Cac)^2)*(1/(1+CSQN_total/(KCSQN+Caer)^2))

PKC(CalciumBD) + Calcium_cyto(bd) <-> PKC(CalciumBD!1).Calcium_cyto(bd!1) konCaPKC,koffCaPKC

PKC(DAGBD) + DAG(pkcbd) <-> PKC(DAGBD!1).DAG(pkcbd!1) konDAGPKC,koffDAGPKC

CIB1(EF1) + Calcium_cyto(bd) <-> CIB1(EF1!1).Calcium_cyto(bd!1) kon1CaCIB1,koff1CaCIB1

CIB1(EF2) + Calcium_cyto(bd) <-> CIB1(EF2!1).Calcium_cyto(bd!1) kon2CaCIB1,koff2CaCIB1

CIB1(EF1!+,EF2!+,sk1bd,location~cytosol) + SphK(CIB1bd,Serk~pS) <-> \

CIB1(EF1!+,EF2!+,sk1bd!1,location~cytosol).SphK(CIB1bd!1,Serk~pS) konCIB1SphK1,koffCIB1SphK1

ERK2(S2~pS) + SphK(Serk~S) -> ERK2(S2~pS) + SphK(Serk~pS) kcatERK/(freeSphK1+kmERKSK1)

CIB1(EF1!+,EF2!+,sk1bd!+,location~cytosol) <-> CIB1(EF1!+,EF2!+,sk1bd!+,location~membrane) ktSK1,ktoffSK1

CIB1(EF1,EF2,location~membrane) -> CIB1(EF1,EF2,location~cytosol) koffSK1

PKC(CalciumBD!+,DAGBD!1).DAG(pkcbd!1) + Raf(Spkc~S) -> \

PKC(CalciumBD!+,DAGBD!1).DAG(pkcbd!1) + Raf(Spkc~pS) kcatPKC/(freeraf+kmPKCRaf)

SphK(Serk~pS) -> SphK(Serk~S) kdpSK1

Raf(Spkc~pS) -> Raf(Spkc~S) kdpPKCRaf

I() -> I() + Sph(skbd) kSphgen

CIB1(sk1bd!1,location~membrane).SphK(CIB1bd!1,Serk~pS) + Sph(skbd) -> S1P(bd) + CIB1(sk1bd!1,location~membrane).SphK(CIB1bd!1,Serk~pS) \

kcatSK1Sph/(KmSK1Sph+freesphingosin)

S1P(bd) -> Sph(skbd) kdpS1P

I() -> I() + RasGTP(rafbd) kS1PRas*frees1p/(KmS1PRas+frees1p)-kRasGAP*gtpfreeras

Raf(rasbd) + RasGTP(rafbd) <-> Raf(rasbd!1).RasGTP(rafbd!1) konRasRaf,koffRasRaf

Raf(rasbd!1,Y1Y2~Y).RasGTP(rafbd!1) -> Raf(rasbd!1,Y1Y2~pY).RasGTP(rafbd!1) kpRaf # Activation of Raf by Tyrosine phosphorylation

Raf(Y1Y2~pY) -> Raf(Y1Y2~Y) kdpRaf

MEK12(S1~S) + Raf(Y1Y2~pY,Spkc~S) -> Raf(Y1Y2~pY,Spkc~S) + MEK12(S1~pS) kpMEK12Raf1/(KmMEK12Raf+mek12s1)

MEK12(S2~S) + Raf(Y1Y2~pY,Spkc~S) -> Raf(Y1Y2~pY,Spkc~S) + MEK12(S2~pS) kpMEK12Raf2/(KmMEK12Raf+mek12s2)

MEK12(S1~S) + Raf(Y1Y2~pY,Spkc~pS) -> Raf(Y1Y2~pY,Spkc~pS) + MEK12(S1~pS) kpMEK12Raf1/(KmMEK12Raf+mek12s2)

MEK12(S2~S) + Raf(Y1Y2~pY,Spkc~pS) -> Raf(Y1Y2~pY,Spkc~pS) + MEK12(S2~pS) kpMEK12Raf2/(KmMEK12Raf+mek12s2)

MEK12(S1~S) + Raf(Y1Y2~Y,Spkc~pS) -> Raf(Y1Y2~Y,Spkc~pS) + MEK12(S1~pS) kpMEK12Raf1/(KmMEK12Raf+mek12s1)

MEK12(S2~S) + Raf(Y1Y2~Y,Spkc~pS) -> Raf(Y1Y2~Y,Spkc~pS) + MEK12(S2~pS) kpMEK12Raf2/(KmMEK12Raf+mek12s2)

MEK12(S1~pS) -> MEK12(S1~S) kdpMEK12_1

MEK12(S2~pS) -> MEK12(S2~S) kdpMEK12_2

MEK12(S1~pS,S2~pS) + ERK1(S1~S) -> MEK12(S1~pS,S2~pS) + ERK1(S1~pS) \

(kpMEK12ERK12_1/(kmMEKERK12+erk12s1))

MEK12(S1~pS,S2~pS) + ERK2(S2~S) -> \

MEK12(S1~pS,S2~pS) + ERK2(S2~pS) \

(kpMEK12ERK12_2/(kmMEKERK12+erk12s2))

ERK1(S1~pS) -> ERK1(S1~S) kdpERK12_1

ERK2(S2~pS) -> ERK2(S2~S) kdpERK12_2

end reaction rules

end model

## actions ##

generate_network({overwrite=>1,max_agg=>10})

writeMexfile({atol=>1e-8,rtol=>1e-8,t_start=>0,t_end=>100000,n_steps=>10000,max_num_steps=>50000,sparse=>1,stiff=>1})

writeSBML({})
